# A genome-wide survey of maternal and embryonic transcripts during *Xenopus tropicalis* development

**DOI:** 10.1186/1471-2164-14-762

**Published:** 2013-11-06

**Authors:** Sarita S Paranjpe, Ulrike G Jacobi, Simon J van Heeringen, Gert Jan C Veenstra

**Affiliations:** 1 Radboud University Nijmegen, Dept. of Molecular Developmental Biology, Faculty of Science, Nijmegen Center for Molecular Life Sciences, The Netherlands; 2 Current address: Teijin Aramid BV, Research Institute QRI, Arnhem, The Netherlands

**Keywords:** *Xenopus tropicalis*, RNA-seq, Maternal and embryonic transcriptome, Polyadenylation, Deadenylation, MBT, Codon bias, Long-noncoding RNAs

## Abstract

**Background:**

Dynamics of polyadenylation vs. deadenylation determine the fate of several developmentally regulated genes. Decay of a subset of maternal mRNAs and new transcription define the maternal-to-zygotic transition, but the full complement of polyadenylated and deadenylated coding and non-coding transcripts has not yet been assessed in *Xenopus* embryos.

**Results:**

To analyze the dynamics and diversity of coding and non-coding transcripts during development, both polyadenylated mRNA and ribosomal RNA-depleted total RNA were harvested across six developmental stages and subjected to high throughput sequencing. The maternally loaded transcriptome is highly diverse and consists of both polyadenylated and deadenylated transcripts. Many maternal genes show peak expression in the oocyte and include genes which are known to be the key regulators of events like oocyte maturation and fertilization. Of all the transcripts that increase in abundance between early blastula and larval stages, about 30% of the embryonic genes are induced by fourfold or more by the late blastula stage and another 35% by late gastrulation. Using a gene model validation and discovery pipeline, we identified novel transcripts and putative long non-coding RNAs (lncRNA). These lncRNA transcripts were stringently selected as spliced transcripts generated from independent promoters, with limited coding potential and a codon bias characteristic of noncoding sequences. Many lncRNAs are conserved and expressed in a developmental stage-specific fashion.

**Conclusions:**

These data reveal dynamics of transcriptome polyadenylation and abundance and provides a high-confidence catalogue of novel and long non-coding RNAs.

## Background

Innovations in sequencing technology have allowed deep sequencing of complementary DNA (cDNA), known as ribonucleic acid sequencing (RNA-seq), enabling transcriptome assembly and identification of coding and non-coding transcripts across many cell types [1-4].

Transcriptome profiling studies have been undertaken in zebrafish, using polyadenylated (polyA^+^) selected messenger RNA (mRNA). These studies have reported identification of thousands of maternal genes and identified the earliest set of embryonic transcripts. They also identified a large number of novel transcribed regions in annotated and unannotated regions of the zebrafish genome [5,6]. In *Xenopus*, several deep-sequencing studies have created different libraries of small RNAs from oocytes, eggs, gastrula, liver and skin [7-9]. A gastrula stage polyadenylated (polyA^+^) selected RNA-seq profile was used to identify transcribed loci, to enhance gene annotation and to analyze spatial regulation of gene expression [10]. Recently, similar polyA^+^ libraries of multiple stages of development were published [11]. For the analysis of transcriptome dynamics it is important to appreciate that, like many other vertebrates, the *Xenopus* maternal-to-zygotic transition involves two important processes: first, destabilization of a subset of maternal mRNAs; second, onset of transcription at the mid-blastula transition (MBT) [12-14]. Studies in *Xenopus laevis* identified distinct phases of maternal, late embryonic and larval gene expression during the course of embryogenesis, whereas microarray analysis in *Xenopus tropicalis* identified several developmentally important maternal mRNAs that are regulated by changes in their adenylation during oogenesis and early development [15,16]. Cytoplasmic polyadenylation is essential for the meiotic maturation of the oocyte as it mediates translational activation of mRNAs encoding *mos* kinase and mitotic cyclins involved in early rapid synchronous cell divisions [17-20]. Several maternally polyadenylated mRNAs lose their polyadenylated tails after fertilization. In most cases, this is mediated by an embryonic deadenylation element (EDEN) in the 3^′^untranslated region (UTR) of the mRNA, which binds embryonic deadenylation element - binding protein (EDEN-BP) [21]. Processes that regulate mRNA deadenylation and degradation are temporally uncoupled. Deadenylated RNAs are as stable as their polyadenylated counterparts until the blastula stage, several hours after fertilization [22].

For developmental analysis it is important to establish the dynamics and scale of maternal transcript destabilization on a genome-wide level and to identify the full complement of embryonic transcripts, including as of yet unannotated and long non-coding RNAs (lncRNAs), the analysis of which will be facilitated by transcriptome profiling using polyA^+^ or total RNA-sequencing. Here, we present results from polyA^+^ and ribosomal RNA depleted total-RNA (RiboZero, RZ) sequencing. Our study distinguishes changes in polyadenylation and abundance, which is critical for the the analysis of early transcript dynamics and proper identification of maternal and embryonically induced transcripts. The embryonic genome shows a gradual cascade of activation, which involves only a third of the number of genes expressed in the oocyte. By expanding and updating our previously published *Xenopus tropicalis* experimentally validated (Xtev) annotation pipeline, we also identified 2,135 new transcripts resulting in a total collection of 29,663 gene models. These new transcripts do not overlap with gene models in the *Xenopus* model organism database (Xenbase) [23]. Using stringent filtering criteria and manual curation, 31 transcripts were identified as “stand-alone” lncRNA transcripts. We characterize these transcripts in terms of exon number, transcript length, conservation and expression pattern during embryogenesis and thus anticipate that our catalogue of coding and long non-coding transcripts will enable more developmental and genomic studies directed towards dissecting their functional roles.

## Results

### Deep sequencing of PolyA^+^ and total RNA libraries

**Figure 1:**
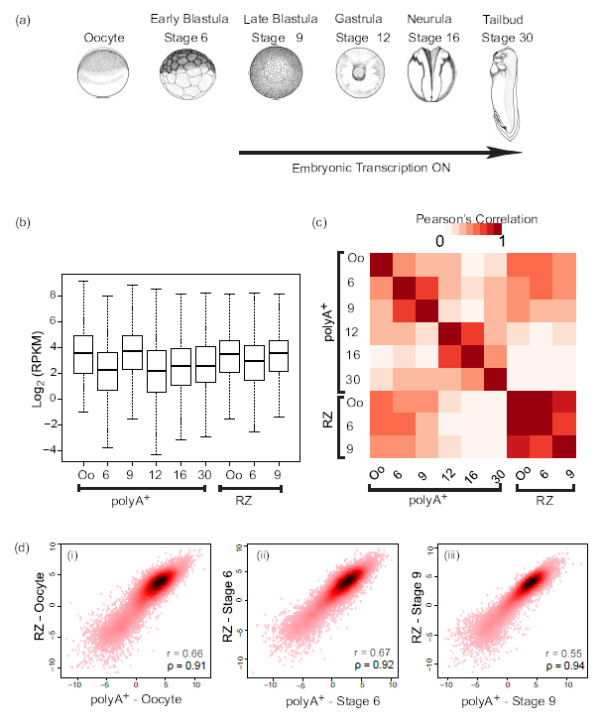
**Generation of RNA-sequencing libraries.(a)** Developmental stages of *Xenopus tropicalis*. **(b)** RPKM distribution across six developmental time-points. Numbers on the x-axis are *Xenopus tropicalis* Nieuwkoop and Faber developmental stages, Oocyte (Oo), stage 6, stage 9, stage 12, stage 16 and stage 30. **(c)** Heat map to show Pearson correlation of expression (RPKM) between all 9 RNA-seq libraries. **(d)** Scatter plots to show stage specific Pearson correlation between RNA-seq data generated using two different methods. Log_2_ RPKM values are plotted on x and y axis respectively. PolyA^+^ (RNA harvested with double PolyA^+^ selection), RZ (ribosomal rRNA depleted-total RNA).

To systematically analyze the transcriptome during early development, we performed polyA^+^ and total RNA (RiboZero, RZ) sequencing experiments across *Xenopus tropicalis* embryogenesis based on three biological replicates (Figure 1a): (1) Oocytes (PA, RZ); (2) early blastula (stage 6; PA, RZ); (3) late blastula (stage 9, after MBT; PA, RZ); (4) gastrula (stage 12; PA); (5) neurula (stage 16; PA) and (6) an early larval stage (stage 30; PA). We verified abundance of transcripts in biological replicates with real time RT- PCR (RT-qPCR) using random hexamers. We not only find minimum variance in transcript abundance among replicates, but also the stage-dependent expression dynamics are similar for the replicates (Additional file 1: Figure S1a). Total RNA was independently extracted for each biological replicate, subjected to polyA^+^ or ribosomal RNA depleted total RNA (RZ) enrichment protocols, quality-controlled, pooled and converted into complementary DNA sequencing libraries for the Illumina Genome Analyzer platform (Additional file 1: Table S1, see Materials and methods).

Gene expression was calculated as reads per kilobase of exon model per million mapped reads (RPKM, see Materials and methods) and shows a comparable median distribution across sequencing libraries (Figure 1b). Heatmap representation of the Pearson correlation coefficients reveal the similarity within the early (oocyte, stage 6, stage 9) and the late (stage 12, stage 16, stage 30) transcriptomes respectively (Figure 1c). There are major changes in the PA transcriptome marking meiotic maturation and fertilization (oocyte, stage 6) and the maternal-to-zygotic transition (stages 6, 9, 12; Figure 1c). The total RNA (RZ) profiles of the early developmental stages correlate relatively well with each other, especially between oocyte and stage 6, most likely due to the presence of stable maternal RNAs and the early embryo being transcriptionally quiescent. Correlation between the stages is higher for total RNA than the polyA^+^ data, most likely due to changes in polyadenylation of maternal mRNA. To rule out any bias in correlation arising from low expression values, we filtered the data for a threshold of 1 RPKM in oocyte (PA and RZ data). The Pearson correlation heatmap of the filtered data shows a similar profile (Figure 1c, Additional file 1: Figure S1b). The correlation between same stages in different data sets (PA and RZ), while moderate, is highly significant (p ≤10^−15^), reflecting the representation of most transcripts in both types of libraries (Figure 1d, Additional file 1: Table S2). A Spearman’s rank order correlation analysis strongly underscores the similarities in the total RNA and polyA^+^ data (Additional file 1: Figure S1c). The data are also in good agreement with previously published ribonucleic acid sequencing (RNA-seq) and microarray data ([11,24], Additional file 1: Figure S2a, b).

### Abundance and polyadenylation state of maternal and maternal-embryonic transcripts

**Figure 2:**
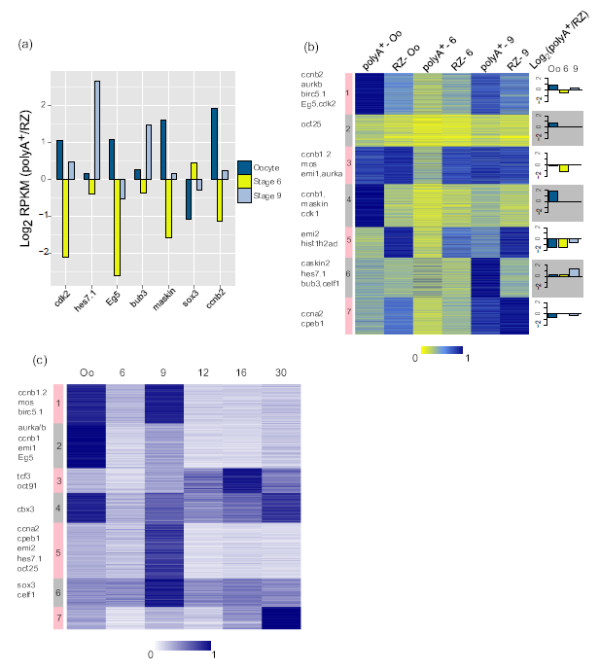
**Total and Polyadenylated RNA profiles of the Maternal Transcriptome.(a)** Barplots to show gene specific distribution of log_2_ RPKM ratios during early development. **(b)** Heatmap to show stage specific comparison between PolyA^+^ and RZ data. The barplots to the right of the figure represent average PolyA^+^ and RZ ratios per stage for the same cluster numbered to the left of the heatmap. Gene names are representative examples from the corresponding cluster. **(c)** Heatmap to show abundance of polyadenylated maternal genes from six developmental time points. Gene names are representative examples from the corresponding cluster. The heatmaps **(bandc)** show scaled expression values (the sum of expression per gene across all stages is set to one). PolyA^+^(RNA harvested with double PolyA^+^ selection), RZ (ribosomal rRNA depleted-total RNA).

To investigate the maternal contribution to the transcriptome of the developing embryo, we used polyA^+^ and total RNA-seq data to classify maternal and embryonic transcripts. A set of 9,513 transcripts were called as maternally expressed as they met a filtering criteria of RPKM greater than or equal to 1 in oocyte (PA or RZ data) (Additional file 2: Page – Maternal genes). This set of transcripts includes maternal-embryonic genes, which are transcribed in both oocytes and embryos. Our maternal subset also includes well known mouse maternal genes like *c-mos*, *zp2*, *zp3* and *SLBP* (for more examples see Additional file 2: Page – Maternal genes) [25,26]. The polyA^+^ and total RNA-seq pool of transcripts are likely to have different complexity, and while their RPKM expression levels cannot be compared directly, the ratio of these two measures (PA/RZ) may reflect the relative polyadenylation state. This would allow us to examine the polyadenylated state of all transcripts during early development. Using this strategy we initially compared the PA/RZ RPKM ratios for genes like *cdk2*(Eg1), *kif11*(Eg5) and *hes7.1*, which are known to be deadenylated after fertilization [15,22], and indeed, their PA/RZ ratios faithfully reflect their fertilization-induced deadenylated state (Figure 2a, Additional file 1: Figure S3a). Genome-wide average PA/RZ ratios show an overall abundance of polyadenylated maternal gene products at stage 9 relative to earlier stages (Additional file 1: Figure S3b). These abundant polyA^+^ transcripts arise either from polyadenylation of the maternally derived message, or new transcription (maternal-embryonic transcripts). We validated the adenylation states of transcripts using RT-qPCR in combination with oligo(dT)_20_ primers and random hexamers (Additional file 1: Figure S3c). With one exception (*sox3*), polyA^+^ ribonucleic acid sequencing (RNA-seq) data and RT-qPCR with oligo(dT)_20_ primers correlated very well. To analyze the deadenylated transcripts more systematically, we filtered the log ratio of two measures (PA/RZ) to be less than or equal to -0.5 for oocyte, stage 6 and stage 9 respectively. This filtering gave us sets of genes that are highly enriched in RZ data compared to the polyA^+^ data (oocyte : 2,675, stage 6 : 5,118, stage 9 : 2,016 genes, see Additional file 2: Page – RZ-enriched genes). Interestingly we find stage 6 to be highly enriched in deadenylated transcripts. This may reflect the fertilization-induced deadenylated state, which has been reported in the literature [27-30]. In order to gain an insight in to the functional categories of deadenylated transcripts at this stage, we compared enrichment of gene ontology (GO) terms of biological processes (BP) using DAVID [31]. We extracted the stage-specific functional annotation charts from DAVID and compared them using clusterProfiler, an R package for comparing gene clusters, with a p-value cut off of 0.01 [32]. Interestingly most of the stage 6 transcripts show an enrichment of biological processes related to cell-cycle control and regulation (Additional file 1: Figure S3d). For example important cell-cycle regulators like *aurka*, *cdc25c* and *birc5.1* belong to stage 6 RZ-enriched fraction. Several chromatin reorganizers and modifiers like *ezh2*, *cbx4* and *hdac3* are also enriched in the stage 6 RZ- enriched fraction.

To assess patterns of genome-wide polyadenylation during early development in more detail, we used K-means clustering (Figure 2b). Clusters 1, 3, and 4 are groups of maternally abundant polyadenylated transcripts and include well known genes like *ccnb1*, *aurkb*, *mos*, *emi1* and *maskin*. These genes show peak expression in the oocyte and are deadenylated or degraded in a stage-specific manner (Additional file 1: Figures S3a and GO term enrichment for cluster 3 - Figure S4a). Cluster 5 represents 12% of all the maternally loaded transcripts which are relatively deadenylated. This cluster includes histone variants like *hist1h2ad*, *hist1h2al*, which are known to exist as deadenylated transcripts [33,34], *emi2* which is well studied for its role in unfertilized eggs where it, along with its partner *mos* causes arrest at metaphase of meiosis II (see GO term enrichment for cluster 5 in Additional file 1: Figure S4a) [35,36]. Cluster 6 transcripts are polyadenylated during early development. A notable gene in this cluster is *celf1*, which codes for embryonic deadenylation element - binding protein (EDEN-BP), known to mediate sequence-specific mRNA deadenylation [37-39]. Cluster 7 represents a group of genes that seem to be loaded as relatively deadenylated messages in the oocyte and are then polyadenylated post-fertilization or post-MBT (see GO term enrichment for cluster 7 in Additional file 1: Figure S4a). Overall 59% (clusters 1,2,3,4) of transcripts are deadenylated during oocyte maturation and early post-fertilization development, whereas 57% (clusters 1,3,5,6) show a higher relative polyadenylation state in late blastulae compared to early blastulae. Motif analysis of 3^′^ ends of transcripts clustered in Figure 2b show a significant enrichment of deadenylation and polyadenylation elements (ARE, EDEN and eCPE) in several clusters (Additional file 1: Figure S4b).

To gain insight into the fate and temporal expression patterns of maternally-abundant polyadenylated transcripts after the blastula stage, we compared the polyA^+^ data from six stages (oocyte, stage 6, stage 9, stage 12, stage 16 and stage 30) using K-means clustering (Figure 2c). Cluster 2 includes *aurkb*, a mitotic serinethreonine kinase, which declines in abundance post-MBT. We find that genes like *tcf3* and *oct91* from cluster 3 have different profiles of abundance during development. *oct91*, a homologue of the mammalian pluripotency factor *oct3/4*, peaks in abundance at late gastrula (stage 12) and declines drastically thereafter (Additional file 1: Figure S5f) [40]. On the other hand *tcf3*, a gene encoding a helix-loop-helix transcription factor responsible for mesoderm and axis formation as well as anterior forebrain development via repression of *wnt/beta-catenin* targets, dramatically peaks at blastula and then exists as a stable polyadenylated transcript up to organogenesis (Additional file 1: Figure S5b) [41-44]. This analysis shows that the abundance of many maternally loaded polyadenylated transcripts declines after late blastula.

### Progressive activation of the embryonic genome

**Figure 3:**
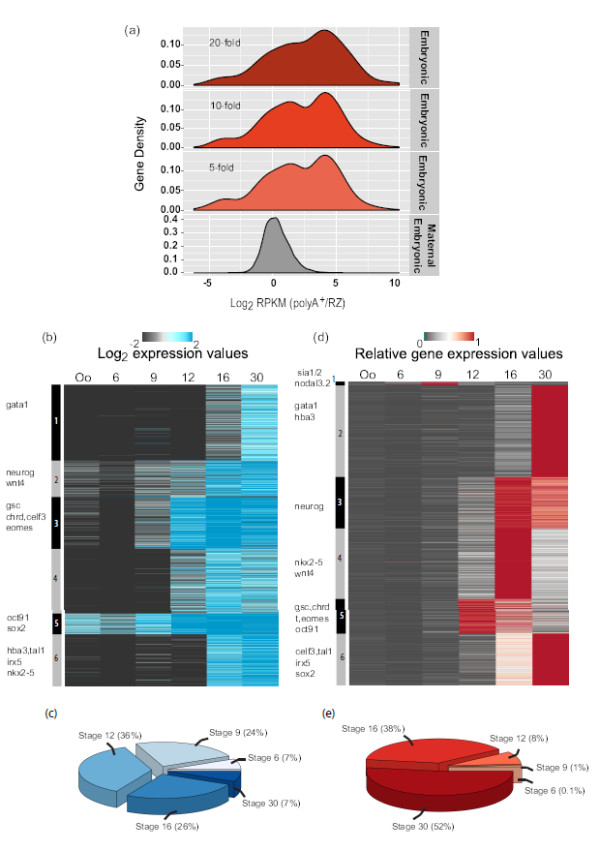
**Overview of the Embryonic Transcriptome.(a)** Density plot to show distribution of Maternal-Embryonic (grey) and Embryonic (red) ratios of polyA^+^ vs. RZ expression (RPKM) at Stage 9. **(b)** Heatmap to show dynamic expression of 2,481 polyadenylated embryonic genes. Scale represents the log_2_ transformed RPKM values. Gene names are representative examples from the corresponding cluster. **(c)** A pie-chart to show percentage of genes whose expression is increased four folds or more relative to Oocyte. **(d)** A heatmap to show scaled expression (the sum of expression per gene across all stages is set to one) of 2,481 polyadenylated embryonic genes. Gene names are representative examples from the corresponding cluster. **(e)** A pie-chart to show percentage of embryonic genes peaking in expression per stage.

An embryonic set of 2,481 transcripts was obtained by filtering the data for a 10-fold increase in any of the stages compared to oocyte expression. This set includes transcripts which are transcribed around or immediately after the mid-blastula transition (MBT). Concomitant polyadenylation could stabilize the newly synthesized transcripts and mark them for translation. To examine whether this is the case we compared the relative adenylation status of embryonic transcripts expressed at stage 9 to that of maternally abundant stage 9 transcripts. This comparison revealed that a large number of stage 9 embryonic transcripts are highly polyadenylated compared to their maternal counterparts, although the polyA^+^ state is also more variable in the embryonic subset of the late blastula transcriptome than in the maternal-embryonic subset (Figure 3a). The polyadenylation distribution of this variable embryonic transcriptome at stage 9 is robust and does not change with filtering criteria of embryonic transcripts (fold increase relative to oocyte levels) (Figure 3a). To explore this variable transcriptomic landscape during embryogenesis, we used K-means clustering to group genes according to their expression (log-transformed RPKM values) or according to their expression relative to maximum gene expression (scaled expression per gene) (Figures 3b and d). This revealed that many genes are activated at the MBT, but most are more strongly induced at later stages (Figure 3b). Comparing the numbers of expressed transcripts relative to the total embryonic pool of transcripts reveals that about 30% of the embryonic genes are induced by fourfold or more during blastula stages and another 36% by late gastrulation (Figure 3c). Clustering gene expression relative to maximum gene expression reveals exquisitely well-defined clusters of dynamic gene expression (Figure 3d). Genes which peak in the late blastula (Figure 3d, cluster 1) include *nodal 3.2/5/6* and *sia1/2*, respectively signalling and transcription factors important for the SpemannMangold organizer (Additional file 1: Figure S5d and g) [45,46]. Gastrula expression represented by cluster 5 contains 8% of genes peaking in expression (Figure 3e). This cluster is dominated by high expression of genes involved in mesendoderm specification and patterning such as *eomes*, *chrd*, *gsc*, and *t* (Additional file 1: Figure S5g). Cluster 3 and 4 shows genes peaking during neurulation and include genes like *neurog*, *pax6*, *nkx2-5*, *wnt4* and *myod*. Cluster 2 and cluster 6 together represent clusters of genes peaking in expression at stage 30 of development and include transcription factors involved in hematopoietic development like *gata1* and *tal1*. *celf3* is an embryonic gene which belongs to the CELF family of genes that code for RNA binding proteins involved in deadenylation of mRNAs. It is known to be exclusively expressed in the nervous system including domains in the brain, spinal cord, optic and otic vesicles [47]. *irx5*, a homeobox transcription factor known for its role in neural patterning also peaks during organogenesis [48]. As the embryonic genome is progressively taking control of development, we found 50% of the developmentally induced genes reach maximum expression late in development (Figure 3e).

**Figure 4:**
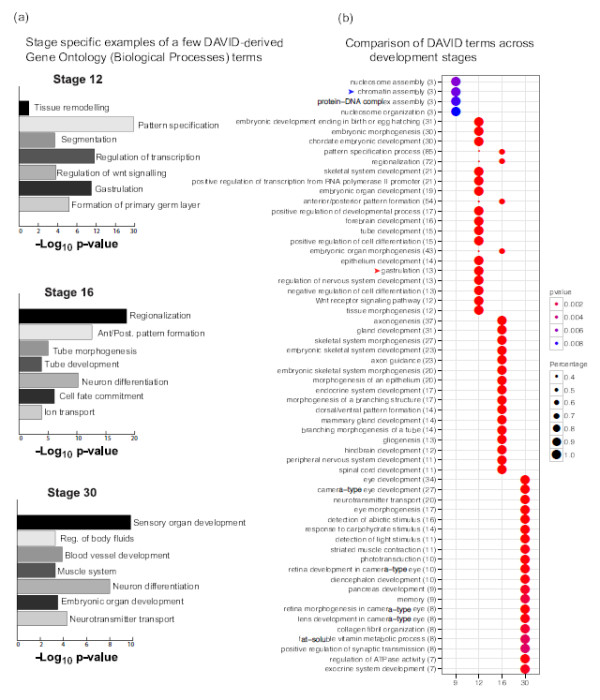
**Gene Ontology Analysis of the Embryonic Transcriptome.(a)** GO term enrichment analysis from DAVID. Barplots (i) Stage 12, (ii) Stage 16, (iii) Stage 30 show stage-specific significant Biological Processes and their -log P-values plotted on x-axis. **(b)** A plot to cluster and visualize DAVID-derived GO terms from developmental stages 9, 12, 16 and 30 using R package clusterProfiler with a p-value cut off < 0.01 [32]. The DAVID GO terms have been derived from biological process annotation of *Xenopus tropicalis* genes.

To correlate these temporal profiles with gene function, enrichment for GO terms of BP were examined using DAVID [31]. Using gene names as unique identifiers, we found a significant enrichment of stage-specific processes (Figure 4a). We then extracted the stage-specific functional annotation charts from DAVID and compared them using clusterProfiler, an R package for comparing gene clusters, with a p-value cut off of 0.01 (Figure 4b) [32]. Cluster 1 (from Figure 3d) is a small cluster of 31 genes and shows enrichment of terms like nucleosome assembly, a term with 3 genes (*hist2h2ab*, *hist1h4k*, *hist1j2aj*) (blue arrowhead, Figure 4b). The stage 12-specific genes (cluster 5, from Figure 3d) show enrichment for the term gastrulation and include well-known genes (for example *bmp4*, *cer1*, *fgf8*, *gsc*, *foxa2*, *nodal*, *eomes*, *lef1*, *lhx1*, *foxc1*, *foxc2*, *chrd*, *gata4*) (red arrowhead, Figure 4b). In conclusion, apart from confirming genes enriched in terms with known functions, our GO analysis also provides a framework of hypothesis for several genes with unknown function.

### Experimental validation of gene models and analysis of novel transcripts

To improve gene annotation and identify potentially novel transcripts, we updated our previously published *Xenopus tropicalis* experimentally validated (Xtev) annotation pipeline [10]. Using more sequencing data and the latest genome build, we performed guided transcript assembly with Cufflinks using all our polyA^+^ and total RNA-seq data with JGI 7.1 annotation as reference [49,50] and combined the Cufflinks transcripts with expressed sequence tags (EST) clusters (Gurdon clusters, courtesy of Dr. Mike Gilchrist). Both histone H3 lysine 4 tri-methylation (H3K4me3) and RNA polymerase II (RNAPII) chromatin immuno-precipitation sequencing (ChIP-seq) data were used to validate or update the 5^′^ ends of the gene models as described previously [10] (see Additional file 1: Figure S6, Additional file 3: Page – Gene models). The annotation pipeline resulted in a collection of 29,663 *Xenopus tropicalis* spliced gene models out of which 18,305 were validated or updated by the *Xenopus tropicalis* experimentally validated (Xtev) pipeline. From these validated models, 17,592 (96%) can be detected by RNA-seq and 65% have H3K4me3 enrichment at the annotated start site. Several thousand gene models were updated and/or reannotated leading to addition of 5^′^, 3^′^ or internal exons (for a complete overview of Xtev(v3.4) known gene model update see Additional file 1: Figures S6 and S7a). In addition 2,135 spliced transcripts were newly identified on basis of RNA-seq and/or EST evidence.

As a by-product of our gene annotation pipeline, we find evidence for a total of 33,601 single exon unspliced gene models. These unspliced single exon gene models are filtered out early on in the pipeline and have not been analyzed further (for a complete list with genomic co-ordinates see Additional file 3: Page – Single exon gene models). These single exon models include MALAT1 (metastasis associated lung adenocarcinoma transcript 1), a known single exon lncRNA conserved in mammals, zebrafish and *Xenopus*[51]. From the expression data it appears to be most abundant at the at neurula stage, suggestive of a specialized stage-dependent regulatory role (Additional file 1: Figure S7b).

**Figure 5:**
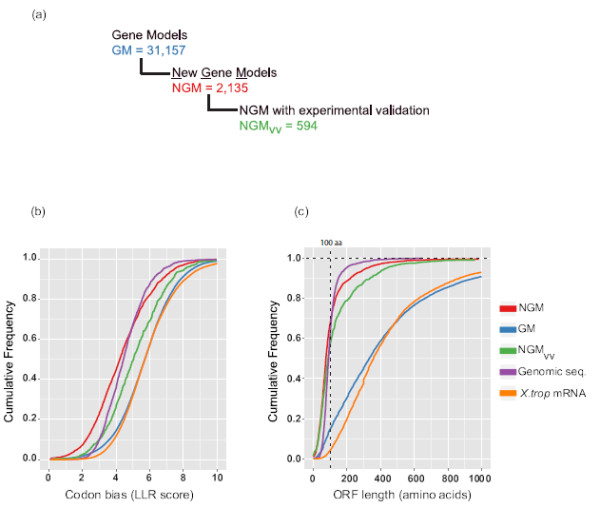
**Analysis of Novel transcripts.(a)** Subsets of gene models from the updated *Xenopus tropicalis* gene annotation pipeline. **(bandc)** Cumulative frequency chart to show distribution of codon bias (LLR score) and ORF length between new gene models (NGM), all gene models (GM), new gene models with validation support (NGM-vv), random genomic sequences (Genomic seq.) and Xenbase extracted *X.tropicalis* mRNAs (*X.trop* mRNA).

Compared to our published annotation pipeline, where we only validated and updated known gene models, which are mostly protein-coding, the new implementation is more inclusive in annotating both coding and long non-coding RNAs. To identify new gene models we looked for Cufflinks transcripts lacking any overlap with gene models from the *Xenopus* model organism database (Xenbase) [23]. We found 2,135 gene models to be new and non-overlapping (new gene models (NGM), Figure 5a, for a complete list with genomic co-ordinates see Additional file 3: Page –NGM). Out of this set, 594 gene models are supported by both expression data (RNA-seq, EST) and a 5^′^ H3K4me3 modification peak (new gene models with validation support (NGM-vv), see Additional file 1: Figure S6, for a complete list with genomic co-ordinates co-ordinates see Additional file 3: Page – NGM-vv), meaning that they are likely stand-alone transcripts. To validate some of these transcripts, we looked at their relative abundance by performing RT-qPCR of RNA from several developmental stages (stage 9, stage 10, stage 10.5 and stage 12). We find stage-specific expression (Additional file 1: Figure S8a).

**Figure 6:**
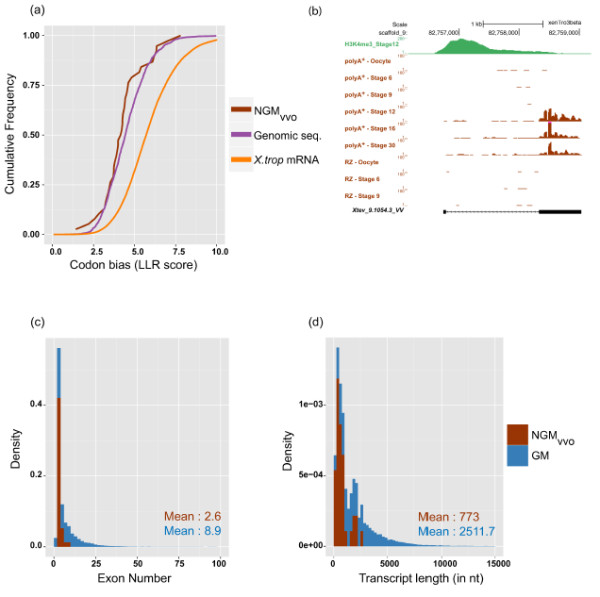
**Analysis of NGM-vvo transcripts.(a)** Cumulative frequency chart to show distribution of codon bias (LLR score) for NGM-vvo, random genomic sequences (Genomic seq.) and Xenbase extracted *X.tropicalis* mRNAs (*X.trop* mRNA). **(b)** An example to illustrate NGM-vvo gene model. H3K4me3 peak demonstrates the gene being transcribed from its own promoter [10]. **(candd)** Frequency distribution to compare number of exons and transcript length (nt, nucleotides) between all gene models (GM) and new gene models (NGM-vvo).

To assess the coding potential of the various gene model subsets (Xtev gene models (GM), NGM, NGM-vv) we used open reading frame (ORF) length and the log likelihood ratio (LLR) of codon bias (see Materials and methods). We find that 65% of new transcripts that are not overlapping with known genes (NGM subset), show a codon bias score comparable to non-coding genomic sequences (Figure 5b). Also, 70% of these NGM transcripts have a maximum ORF length comparable to non-coding genomic sequences (Figure 5c). By contrast, transcripts from annotated protein-coding genes (*Xenopus tropicalis* mRNA) and all GM have large ORF lengths and higher codon bias score, the cumulative distribution of which is well-separated from that of non-coding genomic DNA. However it should be noted that the subset of new non-overlapping transcripts that has strong experimental validation support (594, NGM-vv) may contain both coding and non-coding transcripts. 60% have an ORF length of less than 100 amino acids and also the codon bias distribution suggests this subset represents a mixed population of coding and non-coding RNAs (Figure 5b and c). To enrich for long non-coding RNAs (lncRNAs), we curated the NGM-vv subset both bioinformatically and manually. Since high-confidence lncRNAs have been shown to lack an ORF length greater than 100 amino acids [4], we used this as a first step to enrich for putative lncRNAs. Inspection of this subset of gene models revealed that it was enriched for the 5^′^ untranslated region (UTR) exons of downstream gene models without H3K4me3 peak and a number of other artifacts. We therefore excluded new gene models that were upstream of the known genes without a H3K4me3 peak, or did not meet more stringent expression and splicing criteria (see Materials and methods). The resulting 98 gene models were screened using BLASTN and BLASTP against homology to known protein coding sequences and an array of other problems on the genome browser. For example models in regions with many gaps were excluded. Also, several intronic transcripts were selected against as they may not represent independent transcription units. This screening resulted in a set of 37 transcripts. We compared our different subsets of transcripts with the new transcripts identified by Tan et al., [11]. The Tan study reports the identification of 13,836 novel transcripts, a set of transcripts which collapses to a set of 3,726 unique models that map to the JGI7.1 genome assembly and are not included in the JGI7.1 annotation. Only 122 of these models overlap with the NGM set of 2,135 transcripts. All of the 37 transcripts we selected were identified in the Tan study, however six of these were linked to protein-coding genes in their multi-stage ribonucleic acid sequencing (RNA-seq) data sets. Therefore, our stringent filtering and curation approach has generally enriched for transcripts identified as new gene models in both studies and we conclude that the remaining 31 transcript models are likely stand-alone transcription units. This subset is referred to as NGM-vvo: manually curated new gene models with H3K4me3 peaks and RNA-seq evidence, and a longest ORF length of 100 amino acids. In terms of assessing coding metrics, this set has a codon bias score comparable to random genomic sequences and their cumulative distribution is well separated from known protein-coding mRNA (Figure 6a). An example is shown in Figure 6b (two more examples shown in Additional file 1: Figure S9a). Compared to the protein-coding transcripts, the manually curated new gene models with ORF less than 100 amino acids (NGM-vvo) transcripts have low exon number and relatively shorter transcripts (Figures 6c and d), similar to what has been reported for lncRNAs [52,53]. Based on our manual curation, coding potential metric and gene-structure analysis, we conclude that NGM-vvo subset represents a set of high-confidence lncRNAs.

**Figure 7:**
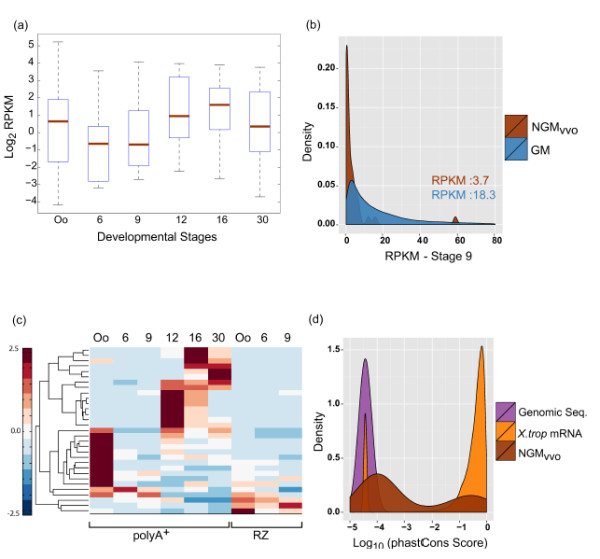
**Expression analysis of putative long non-coding RNAs (NGM-vvo).(a)** Boxplot to show log transformed expression (RPKM, PolyA^+^) across six developmental stages in the NGM-vvo subset. **(b)** Density graph to compare stage-9 expression (RPKM, PolyA^+^) between all gene models (GM) and NGM-vvo subset. **(c)** Heat map to show unsupervised hierarchical clustering of expression (RPKM) of polyA^+^ and RZ data across embryogenesis. Colorscale represents deviation from mean expression calculated row-wise. **(d)** Density plot to compare distribution of log_10_ transformed conservation score (phastCons analysis, see Materials and methods) between random genomic sequences (Genomic Seq), Xenbase extracted *X.tropicalis* mRNAs (*X.trop* mRNA) and NGM-vvo subset. PolyA^+^(RNA harvested with double polyA^+^ selection), RZ (ribosomal rRNA depleted-total RNA).

Mammalian and zebrafish lncRNA show a mean expression varying from a 3-fold to a 10-fold difference from their protein-coding counterparts [53,54]. To investigate the expression of NGM-vvo lncRNAs during embryogenesis, we looked at their polyA^+^ mRNA profiles during embryogenesis. We find that the median expression level of lncRNAs during embryogenesis is one-third of their protein-coding counterparts (Figures 7a and b and Additional file 1: Figure S8b). To investigate expression patterns of NGM-vvo transcripts, we performed unsupervised hierarchical clustering of polyA^+^ and total-RNA expression profiles during embryogenesis. Just like the protein-coding genes, some NGM-vvo transcripts have maternal expression. Many are developmentally regulated and show a stage-specific peak in expression (Figure 7c and Additional file 1: Figures S8b).

The GENCODE v7 catalogue of human lncRNAs has looked into conservation of human lncRNAs using phastCons analysis [52]. Our lncRNA conservation results are in line with these analyses, since we find NGMvvo exons to be less conserved than annotated proteincoding mRNA, but more conserved than the random genomic sequences (Figure 7d). The evolutionary constraints on their sequence and their developmentally regulated expression may be an indication of their stage-specific functionality.

## Discussion

Our results present temporal profiles of maternal and embryonic transcripts during early development. We report a total number of 14,819 non-redundant Xenbase transcripts expressed in any of the six assayed stages from oocyte to tailbud embryos and mapped to *Xenopus tropicalis* genome assembly (Joint Genome Institute (JGI) 7.1). In our data set the maternal transcriptome consists of over 9,000 transcripts that show differential adenylation and of these 46% are abundant in the oocyte polyA^+^data (Figure 2c). This is interesting in view of the fact that the oocyte serves as a reservoir of stable maternal transcripts which drive early development in the absence of embryonic transcription. To better understand the dynamics of polyadenylated vs. deadenylated mRNA, we compared the ratio of polyA^+^ and total RNA. This comparison gave us a tool to examine the dynamics of transcript abundance and polyadenylation during early development. We observed fertilization-induced deadenylation of several cell cycle regulators like *cdk2*(Eg1), *kif11*(Eg5) as already reported [16]. Also, it is interesting to note that there is an exclusive pool of relatively deadenylated transcripts, which in our analysis accounts for 12% of the maternal genes and includes well known non-adenylated transcripts like the histone mRNAs (Figure 2b). Transcription has been reported to start at the mid-blastula stage [12,13,55], although a number of genes are transcribed before this stage. We find little evidence of pre-MBT transcription at stage 6 in our data. Many maternal transcripts are gaining polyadenylation during post-fertilization development and may appear as false-positives in an analysis of early transcription if only polyA^+^ messages are considered. Between stage 6 and stage 9, some genes are activated early as described for several nodal genes [56]. The embryonic transcript abundance is stage-specific. About 30% of the embryonic genes are induced four-fold or more by late blastula and another 35% by late gastrulation (Figure 3c). 50% of the genes peak late in development (Figure 3e). Our GO analysis of embryonic genes provides confirmation of genes with known functions as well as provides a framework for hypothesis for several genes with unknown functions (Figure 4b).

We have generated an updated annotation pipeline for *Xenopus tropicalis* experimentally validated (Xtev) gene models, featuring a total of 31,157 transcripts. Of these, we find 2,135 gene models to be new, however many of these may be linked to known transcripts and are not independently generated. The NGM-vvo subset of 31 transcripts is a high-confidence set of lncRNAs, which shares many of the characteristic features of lncRNAs such as low exon number, relatively short length and overall low expression during embryogenesis [4,51,53]. They are decorated with H3K4me3 histone modification at the 5^′^ end, evidence that these high-confidence lncRNAs are transcribed from their own promoter. Like their protein-coding counterparts, the expression profile of high confidence lncRNAs (NGM-vvo) is stage-specific and temporally restricted.

It proved surprisingly difficult to identify this high-confidence set of lncRNAs. This is because any selection for novel or unannotated transcripts enriches resulting subsets for annotation problems (broken genes) and assembly problems (poorly assembled regions with fragmented genes). Our high-confidence approach may however underestimate the true number of lncRNAs that are expressed during embryogenesis. First of all, lncRNA may be transcribed from complex loci and not all may meet our criteria of stand-alone transcripts. Second, many lncRNAs are expressed at very low levels. Inclusion of more RNA-seq data is therefore likely to identify more lncRNAs. On the other hand, true stand-alone lncRNA transcription units, produced from their own promoter, may be far less common than frequently assumed, and the majority of “new transcripts” may arise as by-products of known genes or be transcribed from highly complex loci. Also, RNA-seq alignment tools produce artifacts and multiple, sometimes incorrect, models for the same locus. Therefore, approaches that integrate expression and histone modification data are essential to curate transcription units.

Functional analyses of the novel lncRNAs are required to elucidate their potential roles in pre-MBT transcriptional repression, gastrulation, neurulation and organogenesis. Our catalogue of high confidence stand-alone lncRNAs with sequence conservation and stage-specific expression provides a prioritized resource for studies in lncRNA function during vertebrate development.

## Conclusions

We provide a comprehensive survey of the *Xenopus tropicalis* transcriptome using polyA^+^ and ribosomal-RNA depleted total RNA expression data. These results provide insights into the maternal and embryonic components of expression and polyadenylation dynamics through-out early embryogenesis. In addition, our improved annotation has led to the discovery of new transcripts which constitute subset of high-confidence stand-alone lncRNAs. Together, these data provide a rich developmentally relevant resource, integration of which will enable new genomic and genetic studies in the near future.

## Materials and methods

### Animal procedures

*X.tropicalis* embryos were obtained with *in vitro* fertilization from three separate crosses (different outbred animals). Briefly, both females and males were primed with 10 units of human chorionic gonadotropin (hCG-pregnyl, Organon). Four to six hours before embryo collection, female frogs were injected with 200 units of hCG. Forty-five minutes after the onset of egg laying, embryos were collected and dejellied in 3% cysteine hydrochloride (pH 8.0) in 10% MMR. Embryos were then cultured in 10% MMR at room temperature and were staged according to Nieuwkoop and Faber (1994) [57]. Embryos from three separate clutches were harvested and frozen at -80°C until RNA isolation. Stage VI oocytes were harvested by treating ovarian follicles with collagenase (*Clostridium* type I collagenase, Sigma).

### RNA preparation and sequencing

Oocyte and embryos from Nieuwkoop-Faber stages 6 - 30 were collected and total RNA was isolated using Trizol and the QIAGEN RNeasy Kit. Subsequently, polyadenylated RNA was selected by enriching with the Oligotex mRNA kit (QIAGEN). To ensure complete removal of ribosomal RNA (rRNA), polyadenylated mRNA was subjected to an additional round of Oligotex treatment. Total RNA was subjected to depletion of ribosomal RNA (rRNA) using Ribozero Epicenter low input kit. Two important quality control measures were taken to confirm removal of ribosomal RNA (rRNA). First, the ribosomal RNA (rRNA) -depleted sample was tested on a RNA-chip (Experion, BIORAD) in comparison with non-depleted total RNA. Absence of 28S and 18S peaks in the ribosomal RNA (rRNA) depleted sample confirmed good depletion. Second, RT-qPCR with primers against 28S, 5S and GAPDH was performed. 28S RNA levels were less than 5%, typically around 1% after depletion, whereas GAPDH was typically at more than 80% of the levels before depletion. For sequencing, cDNA was prepared for both polyA^+^ and RZ samples with random hexamer primers using Superscript III (Invitrogen) and the second strand was made with DNA polymerase I, DNA ligase and T4 DNA polymerase. The purified double-stranded cDNA was used for Illumina sample preparation. All quality control qPCR reactions were performed on a MylQ single-color reader real-time PCR detection system (BioRad) using iQ SYBR Green Supermix (BioRad).

The three biological replicates were checked by RT-qPCR and pooled for sample preparation and sequencing. These samples were then processed according to the manufacturer’s protocol (Illumina). Shortly, adapter sequences were linked to the complementary DNA (cDNA) samples, the library was size selected (300-350bp), and amplified by polymerase chain reaction (PCR). The subsequent sequencing was carried out on Genome Analyzer (Illumina).

### RNA-seq expression analysis

On average, we obtained about 16-50 million reads per stage (Additional file 1: Table S1). Out of the total reads about 50-60% could be aligned to the genome assembly (JGI 7.1) of the *Xenopus tropicalis* genome sequence. To allow a quantitative comparison all reads were normalized before analysis. The transcript list contains all the genes that are expressed (= non zero RPKM) in at least one stage. The RPKM per gene is the mean of all RPKM of all the non-redundant exons of all isoforms per gene. The total list contains around 15,289 genes of which only 470 are not detected as expressed in any stage. All unknown/unnamed gene names have been changed to include the genomic position for reasons of identification. Alignment was performed using Burrows-Wheeler Aligner (BWA), reads mapping to multiple positions (non-unique) were not included in the RPKM calculation [58].

### Xtev (v3.4) gene annotation pipeline

Gene models (JGI 7.2) were downloaded from Xenbase (http://www.xenbase.org) and EST clusters mapped to the JGI 7.1 *X.tropicalis* genome were supplied by Mike Gilchrist (NIMR). Spliced transcription units were generated from the RNA-seq data. All reads were mapped to JGI 7.1 using TopHat v2.0.4 [50]. The TopHat output was filtered to keep only new splice sites with evidence of at least 5 spliced reads. The filtered TopHat output was used with Cufflinks v1.3.0 to perform transcript assembly [49]. The experimental annotation pipeline consists of several steps: 1) collect gene models; 2) update with experimental data; 3) validate and/or update gene models with RNA-seq data; 4) validate and/or update transcription start-site (TSS) with H3K4me3 and/or RNAPII ChIP-seq data and 5) Choose the most likely model (Additional file 1: Figure S6). All Xenbase gene models sharing at least 1 exon were considered as multiple models of a single gene. The EST clusters and transcripts determined by Cufflinks were used to update the gene models with extra putative exons, mainly at the 5^′^ and 3^′^ end of genes. The number of RNA-seq reads was determined for all exons of all models. If 1/3 of the exons of a model contained at least 3 RNA-seq reads the model was considered as expressed. If the first exon of a gene model overlapped with a H3K4me3 peak, the TSS was considered as validated. If no single model of a gene had a validated TSS, we looked for evidence of a TSS upstream. In this case there had to be a H3K4me3 peak upstream of a gene model, with no different gene models in between, and the mean RNAPII level of the region between the upstream H3K4me3 peak and the 5^′^ exon of the gene had to be at least 0.5 of the mean RNAPII level of the gene body. Furthermore, all gene models were checked for evidence of a downstream H3K4me3 peak, which can indicate a putative alternative TSS. For each single gene the most likely model was chosen according to the following criteria, in order of decreasing importance: a validated TSS, number of expressed exons, number of exons. Of the new models, which are not present in the Xenbase JGI 7.2 annotation, only spliced transcripts were included.

### Analysis of coding potential

Coding potential of RNA sequences was determined using maximal ORF length and codon bias metrics, as described [59]. The codon bias metric is based on unequal usage of synonymous codons. Briefly, triplet frequencies were determined in non-coding genomic *X.tropicalis* DNA (JGI4.2, GL172663:1-4,425,020, UCSC table browser basepair-wise intersection of complemented Human Proteins with UCSC xenTro3 assembly), whereas *X.tropicalis* codon frequencies were downloaded from http://www.kazusa.or.jp/codon/cgi-bin/showcodon.cgi?species=8364. Log likelihood ratios (LLRs) for each codon were calculated based on the codon frequency conditional of the encoded amino acid, such that for each codon i coding for amino acid a_*i*_, LLR_*i*_ = log_2_(c_*i*_/n_*i*_), in which c_*i*_ and n_*i*_ correspond to the likelihood of codon i conditional on amino acid a_*i*_ in coding and non-coding sequences respectively (Additional file 4). The total LLR score is determined by summing LLR_*i*_ values in all 90 bp windows in six potential reading frames. After computing a score for windows, the max LLR score was defined as the maximum score observed in all windows of the transcript.

### Quantitative RT-qPCR for known and new gene model (NGM subset) validation

Validation of known and novel transcripts was performed on total-RNA which was subjected to depletion of ribosomal RNA (rRNA) using Ribozero Epicenter low input kit. Total RNA was then DNase treated and column purified to remove any contaminating genomic DNA. cDNA was prepared with oligo(dT)_20_ primers or random hexamers using Superscript III (Invitrogen). qPCR reactions were performed on a MylQ single-color reader real-time PCR detection system (BioRad) using iQ SYBR Green Supermix (BioRad). With Stage 9 as reference, fold change was calculated by normalizing Ct values in Stages 10, 10.5 and 12 against *odc1* gene using the 2^−*ΔΔCt*^ method (for primer sequences see Additional file 5) [60].

### Bioinformatic and manual curation of NGM-vv subset

NGM-vv subset is collection of 594 new gene models. As a first step, we filtered these models for ORF length less than or equal to 100 amino acids. This resulted in a set of 331 gene models, which were then screened using following criteria: 1) Absence of a downstream gene (same orientation) with a X or U annotation for H3K4me3 (see flowchart for Xtev pipeline in Additional file 1: Figure S6); 2) RPKM of all exons should be greater than or equal 1 (to filter out models where our data does not support the model) and 3) Evidence of splicing in our data. This resulted in a set of 98 gene models. These models were then manually curated using BLASTN and BLASTP to filter against homology to known protein coding sequences (as described in the main text).

### Conservation (phastCons) analysis

For conservation analysis all gene models were mapped to JGI v4.1 (UCSC: xenTro2) using blat. The average phastCons score per gene model was calculated using the Conservation track (phastCons7way) of the UCSC genome browser.

### Data Availability

The data have been deposited in *NCBI*^′^*s* Gene Expression Omnibus [61] and are accessible through GEO Series accession number GSE43652. Xtev gene models are available at: http://veenstra.ncmls.nl/genomedata.asp.

### Statement on animal use

Animal care and use was in accordance with national and European guidelines and standard operating procedures approved by the institutional animal care and use committee (Dierexperimentencommissie, DEC).

## Abbreviations

polyA+: Polyadenylated; RT-qPCR: Real time RT-PCR; cDNA: Complementary DNA; RNA-seq: Ribonucleic acid sequencing; mRNA: Messenger RNA; MBT: Mid-blastula transition; EDEN: Embryonic deadenylation element; EDEN-BP: Embryonic deadenylation element - binding protein; UTR: Untranslated] region; Xtev: Xenopus tropicalis experimentally validated; lncRNAs: Long non-coding RNAs; ORF: Open reading frame; PA: PolyA+; RiboZero, RZ: Ribosomal RNA depleted total-RNA; RPKM, see Materials and methods: Reads per kilobase of exon model per million mapped reads; GO: Gene ontology; BP: Biological processes; EST: Expressed sequence tags; GM: Gene models; NGM: New gene models; NGM-vv: New gene models with validation support; NGM-vvo: Manually curated new gene models with ORF less than 100 amino acids; LLR: Log likelihood ratio; JGI: Joint Genome Institute; rRNA: Ribosomal RNA; BWA: Burrows-Wheeler Aligner; PCR: Polymerase chain reaction; H3K4me3: Histone H3 lysine 4 tri-methylation; RZ: Ribosomal RNA depleted total RNA; RNAPII: RNA polymerase II; ChIP-seq: Chromatin immuno-precipitation sequencing; TSS: Transcription start-site.

## Competing interests

The authors declare that they have no competing interests.

## Authors’ contributions

SSP, UGJ and GJCV conceived and designed the study, UGJ performed the RNA-seq experiments, SSP did the analysis and performed the validation experiments. SJvH performed transcriptome assembly, designed and assembled the *Xenopus tropicalis* experimentally validated (Xtev) pipeline and performed the conservation analysis. GJCV performed ORF length and codon bias analysis. SSP and GJCV wrote the manuscript. All authors read and approved the final manuscript.

## Supplementary Material

Additional file 1 Supplementary tables (S1 and S2) and figures (S1-S9). Click here for file

Additional file 2 **Multi-page list of genes expressed in** ***Xenopus tropicalis*.** Click here for file

Additional file 3 Multi-page Xtev annotation pipeline gene list with genomic co-ordinates. Click here for file

Additional file 4 Single-page list of codon usage frequencies used for calculating codon bias score. Click here for file

Additional file 5 Single-page list of primers used for real-time qPCR validation of highly conserved lncRNAs from the NGM subset. Click here for file
