# Supplementary material for: A genome-wide survey of maternal and embryonic transcripts during Xenopus tropicalis development
Source: BMC Genomics. 2013 Nov 6;14:762. doi: 10.1186/1471-2164-14-762 (PMC3907017; doi:10.1186/1471-2164-14-762)
Supplement: Additional file 1 — Supplementary tables (S1 and S2) and figures (S1-S9). [file 1471-2164-14-762-S1.pdf]

Table S1 : Statistics for RNA-sequencing libraries.

**Poly(A) Library**	**Oocyte**	**Stage 6**	**Stage 9**	**Stage 12**	**Stage 16**	**Stage 30**
Total reads	31523769	50019507	16649345	30313122	18261070	19901121
Mapped reads	19659282	16003974	10756981	14999462	11076320	13606315
% mapped	62.4	32.0	64.6	49.5	60.6	68.3

**Ribozero Library**	**Oocyte**	**Stage 6**	**Stage 9**
Total reads	30420177	31831783	30896600
Mapped reads	15064112	13836284	15960698
% mapped	49.5	43.5	51.7

Table S2 : Pearson correlation coefficients for polyA$^+$ vs. total RNA (RZ) libraries.

**Library**	**Oocyte(RZ)**	**Stage 6(RZ)**	**Stage 9(RZ)**
Oocyte(PolyA)	0.66	0.67	0.60
Stage 6(PolyA)	0.65	0.67	0.57
Stage 9(PolyA)	0.56	0.55	0.55
Stage 12(PolyA)	0.40	0.35	0.43
Stage 16(PolyA)	0.36	0.35	0.37
Stage 30(PolyA)	0.35	0.35	0.30

Figure S1

(a) Barplots showing RT-qPCR validation in biological replicates.

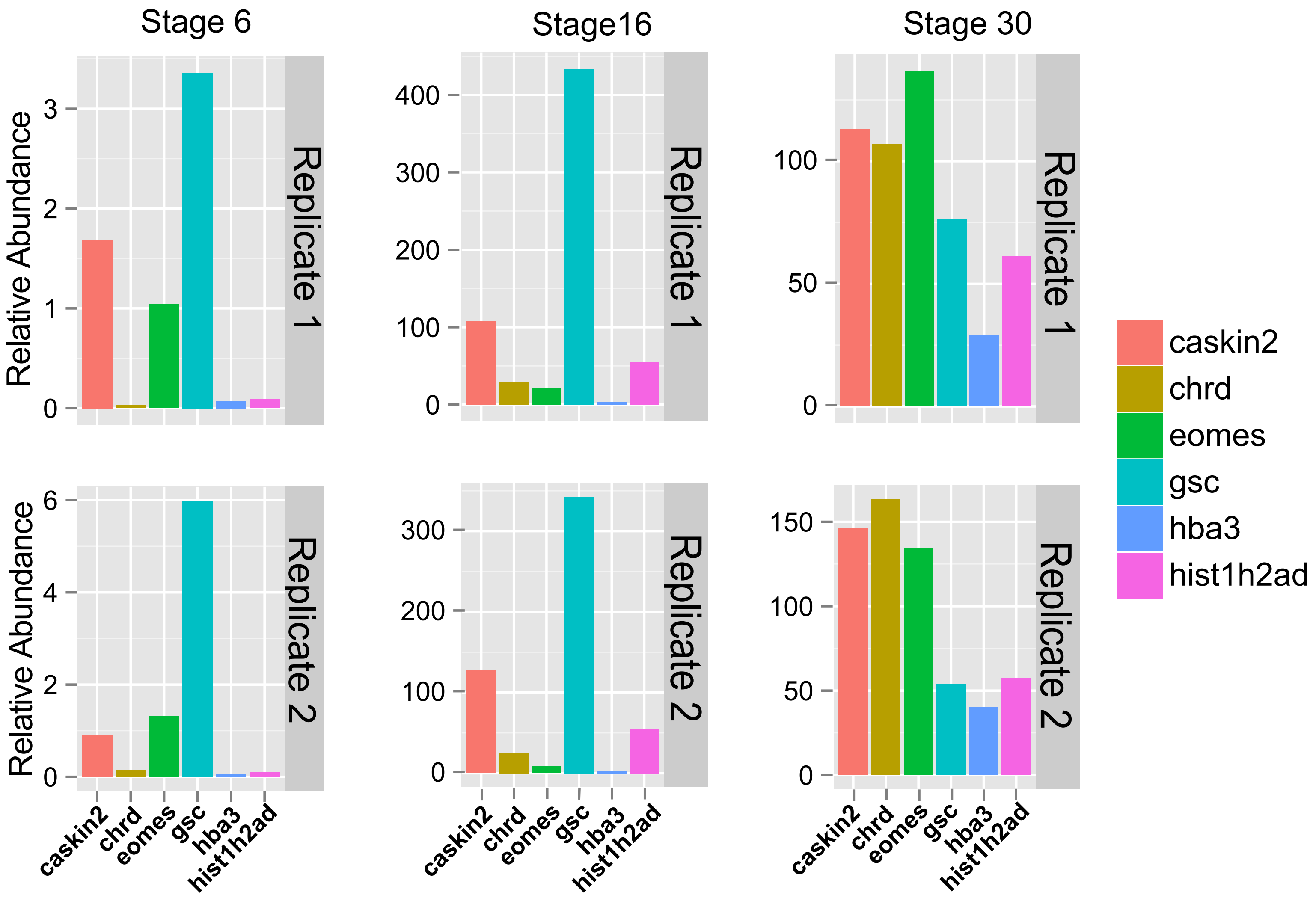

(b) Heatmap showing Pearson correlation between RNA-seq libraries.
Filter : Max [Oocyte polyA+, RZ] RPKM > 1

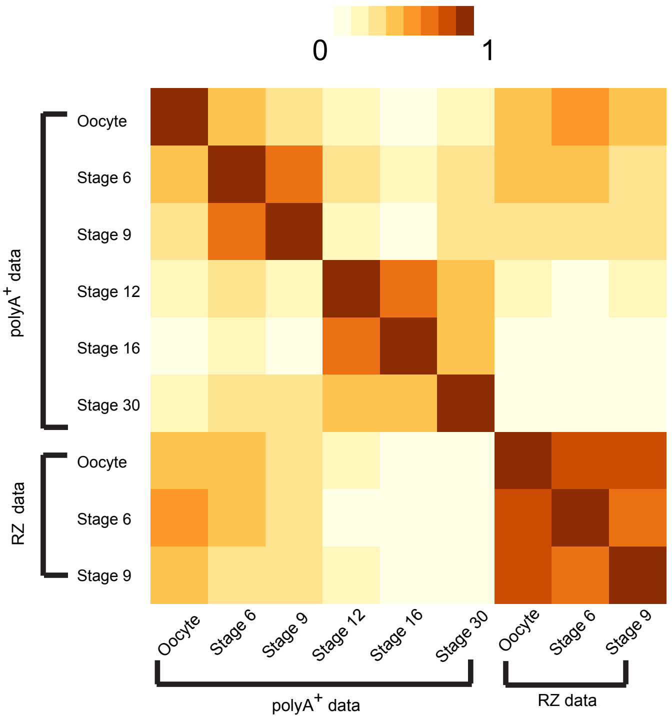

(c) Spearman's rank correlation heatmap.

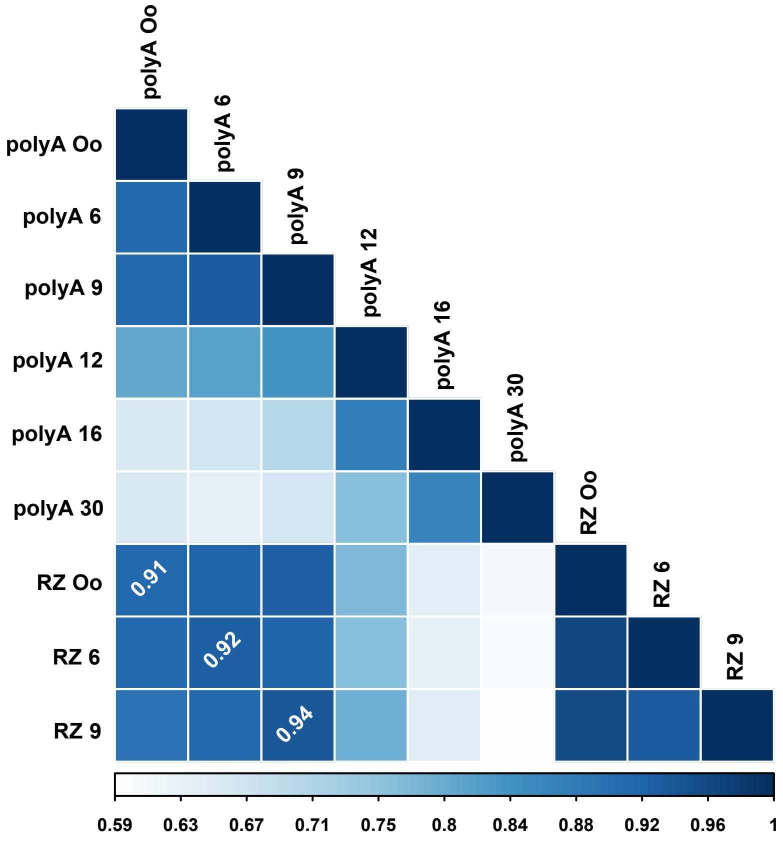

Figure S2

(a) Spearman's rank correlation with published data : Tan MH et al., Genome Res. 2013

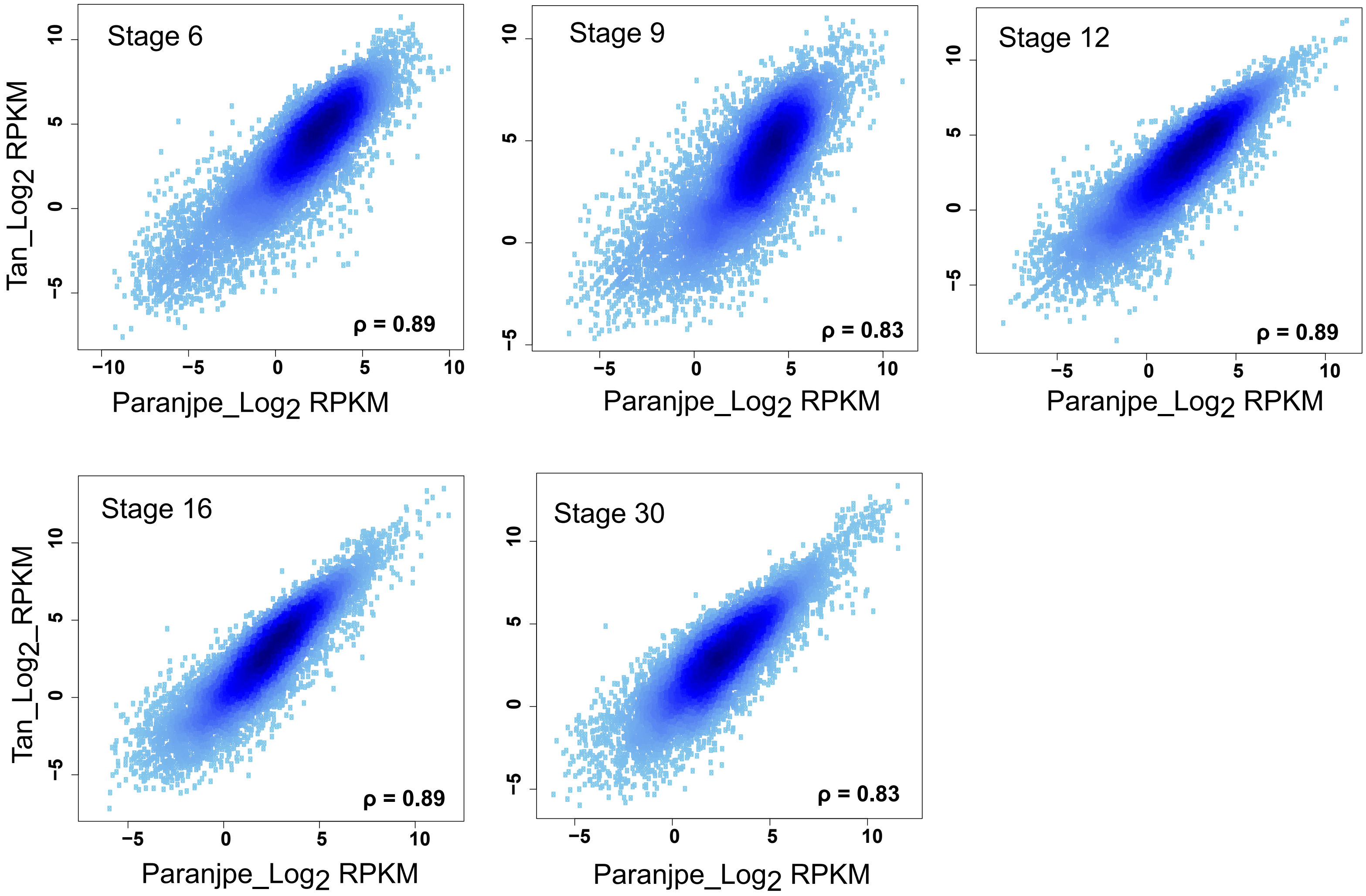

(b) Heatmap to visualize Spearman's rank correlation with published data.

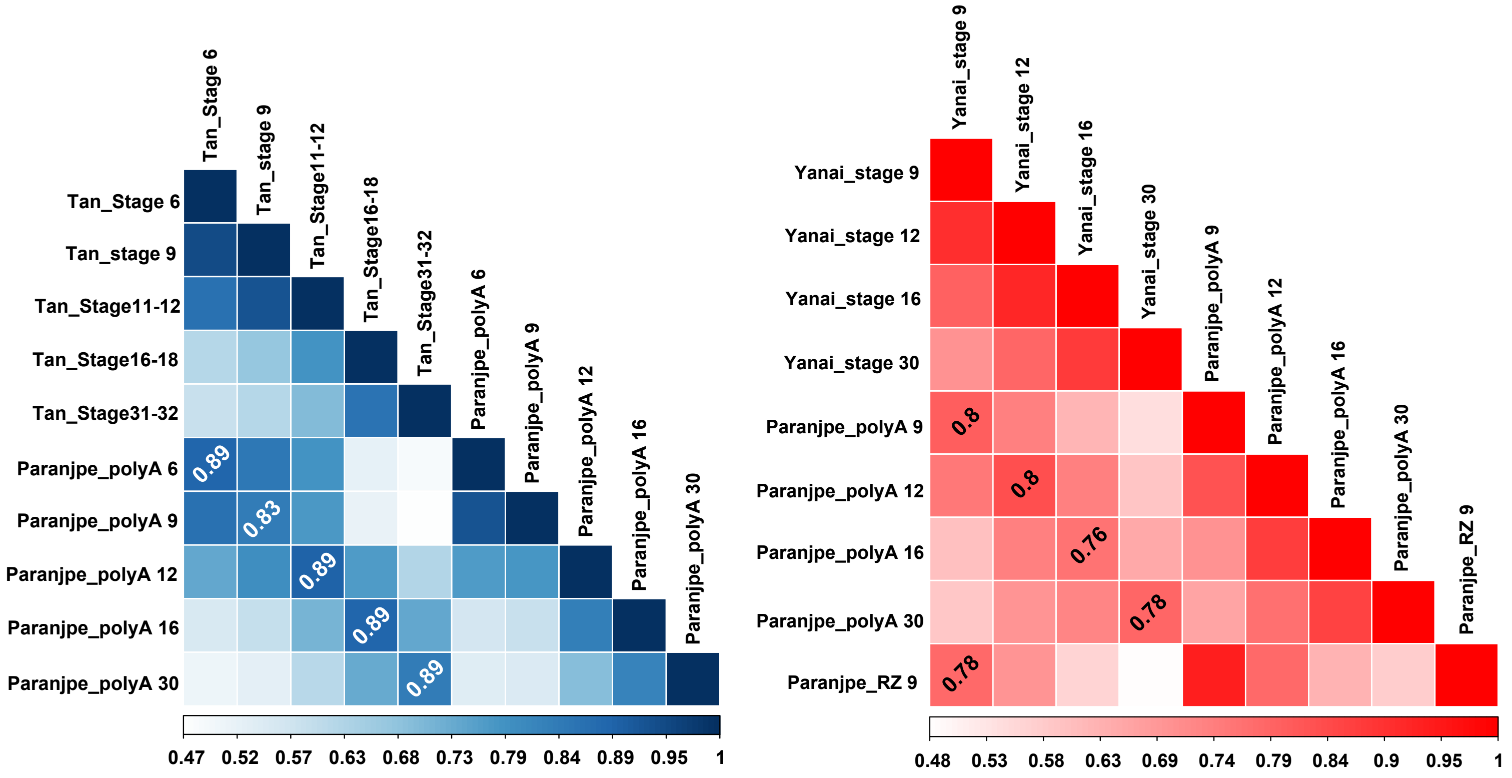

RNA-seq
Tan MH et al., Genome Res. 2013

Microarray
Yanai et al., Dev Cell. 2011

Figure S3

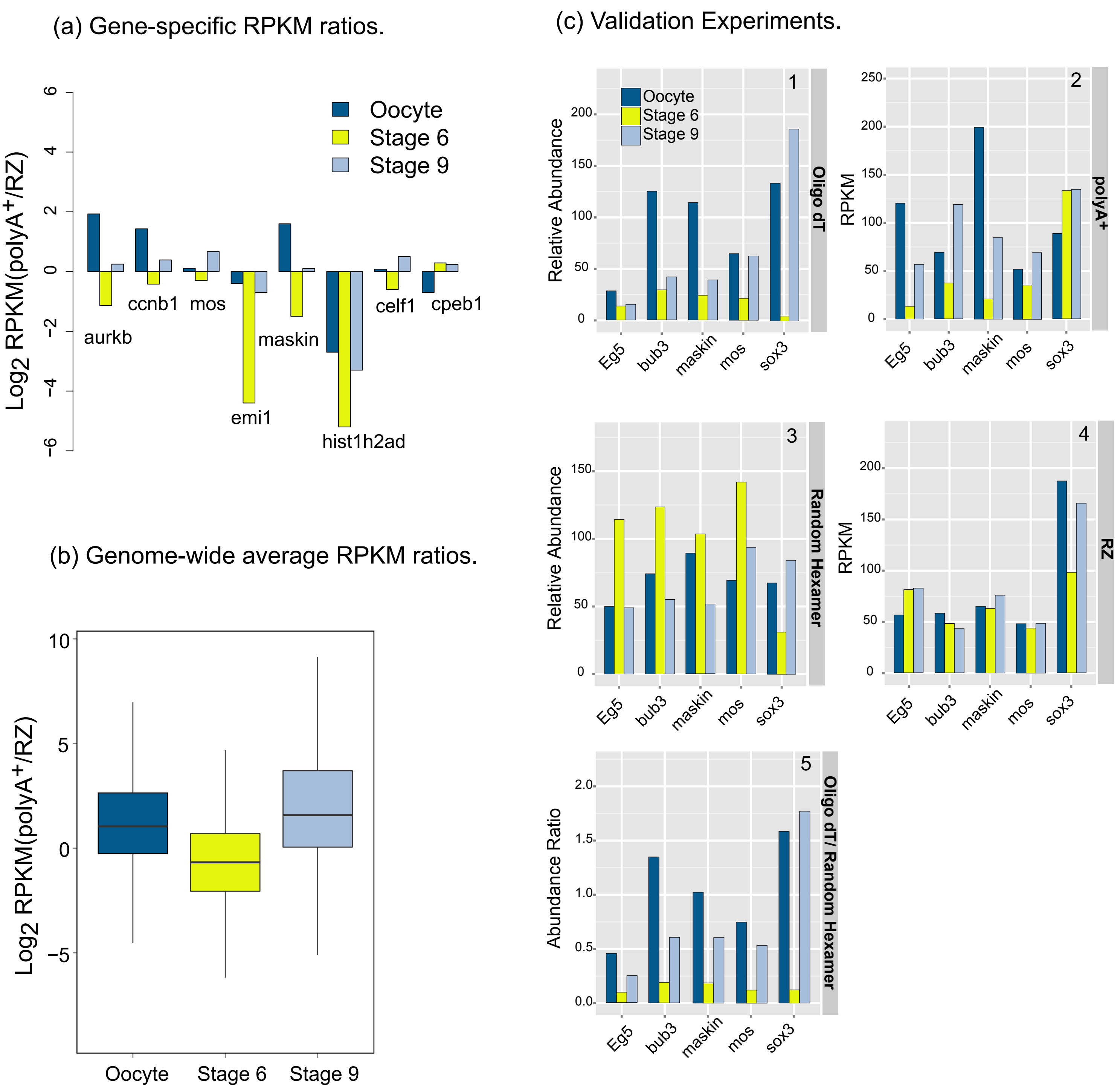

(a) Gene-specific RPKM ratios.
Oocyte
Stage 6
Stage 9
Log2 RPKM(polyA+/RZ)
aurkb
ccnb1
mos
emi1
maskin
hist1h2ad
celf1
cpeb1
(b) Genome-wide average RPKM ratios.
Log2 RPKM(polyA+/RZ)
Oocyte
Stage 6
Stage 9
(c) Validation Experiments.
Relative Abundance
RPKM
Abundance Ratio
Oligo dT
polyA+
Random Hexamer
RZ
Oligo dT/ Random Hexamer
Eg5
bub3
maskin
mos
sox3

Figure S3

(d) Comparison of DAVID-derived GO terms for RZ-enriched genes in Oocyte, Stage 6 and Stage 9 respectively.

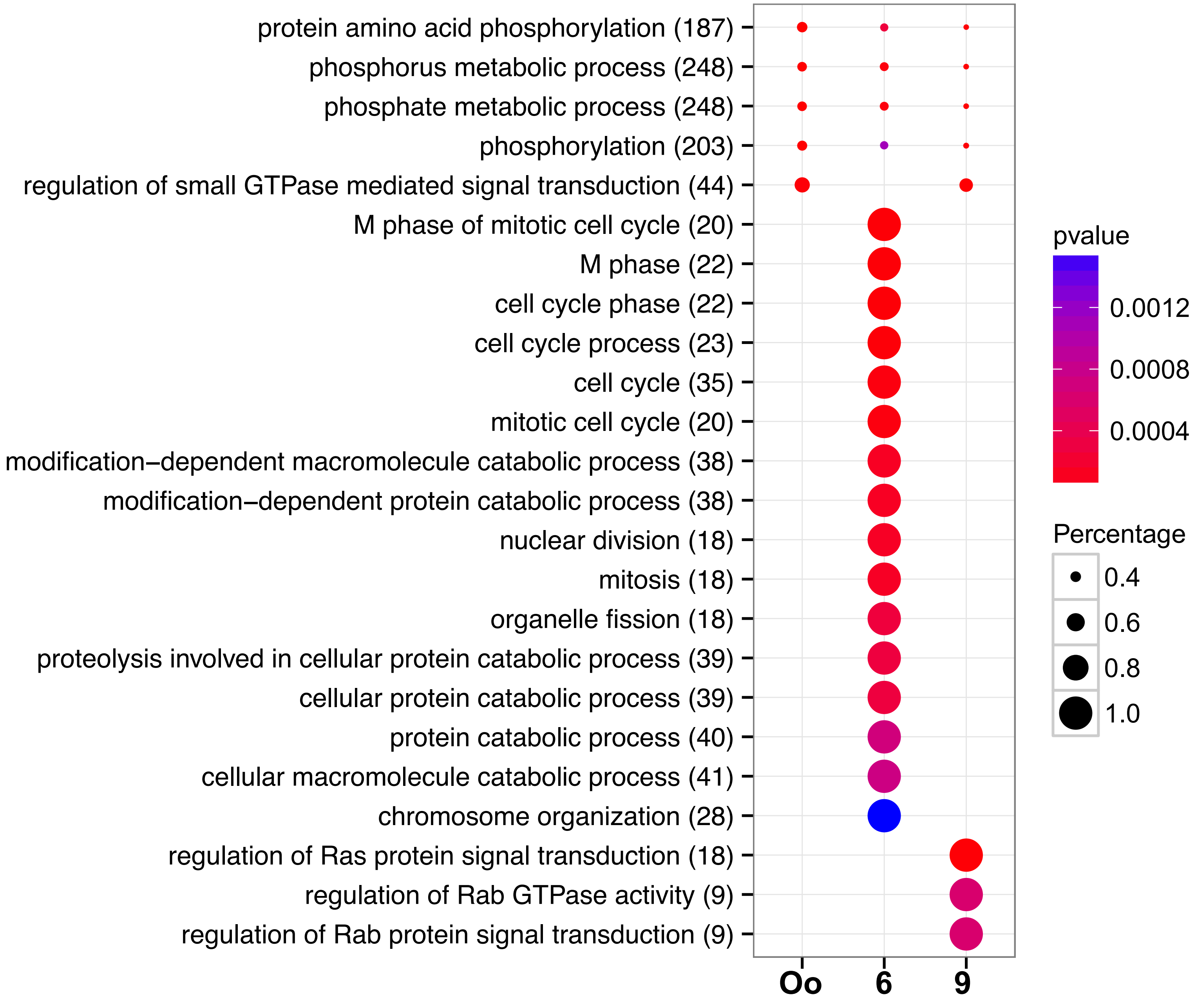

Figure S4

(a) Comparison of DAVID-derived GO terms for clusters 1-7 related to Figure 2b.

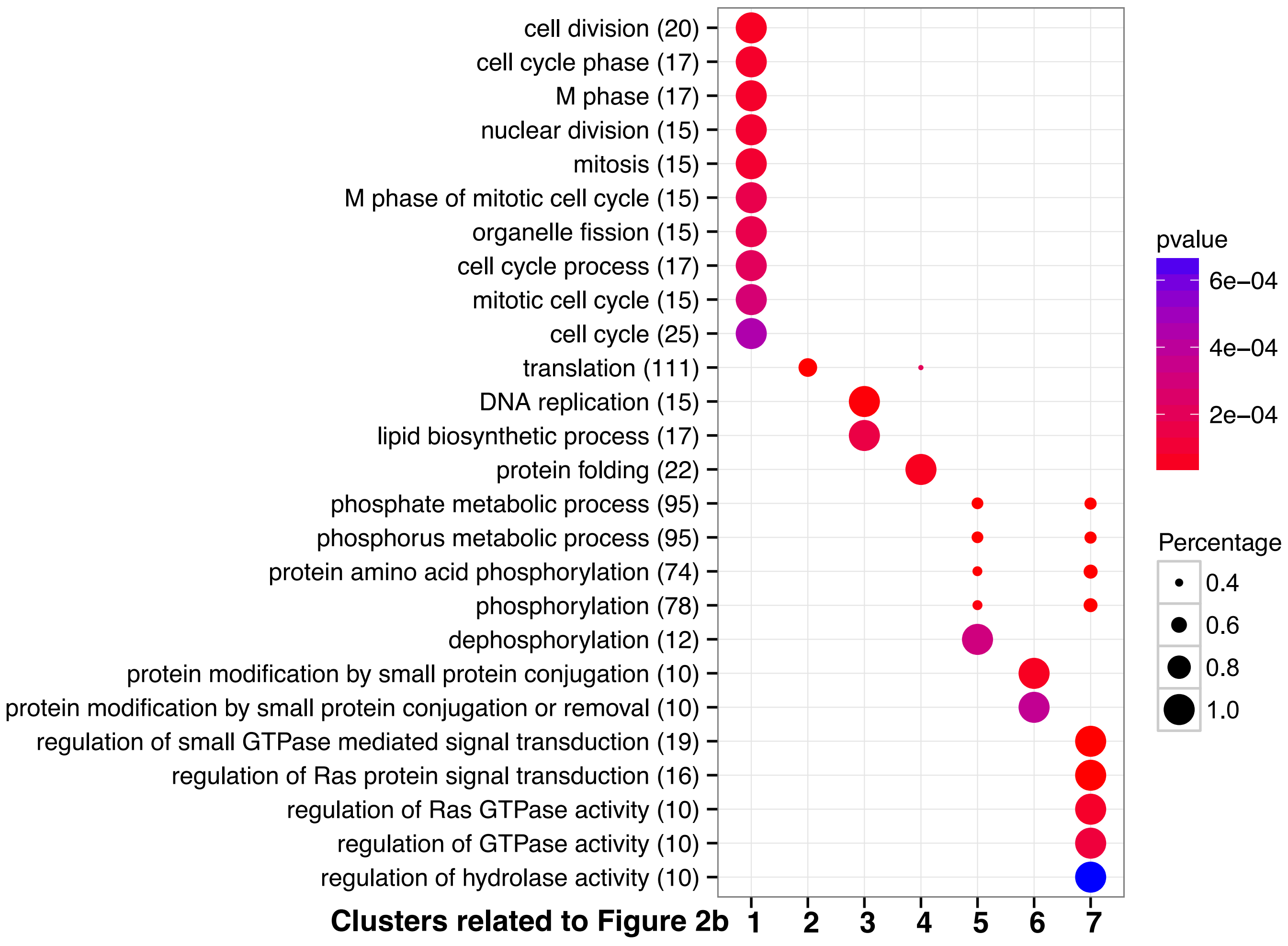

(b) A heatmap to visualize adenylation motif enrichment in clusters 1-7 related to Figure 2b.
(ARE : A-U rich elements, CPE : Cytoplasmic polyadenylation element,
EDEN : Embryonic deadenylation element, eCPE : Embryonic -CPE)

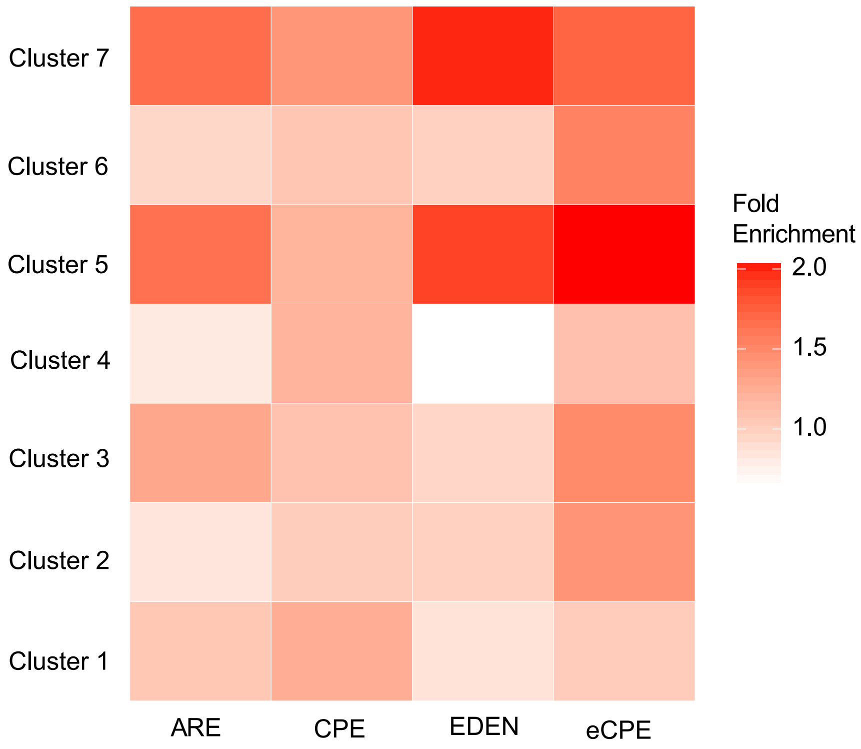

Figure S5. UCSC Genome browser shots of developmentally regulated genes

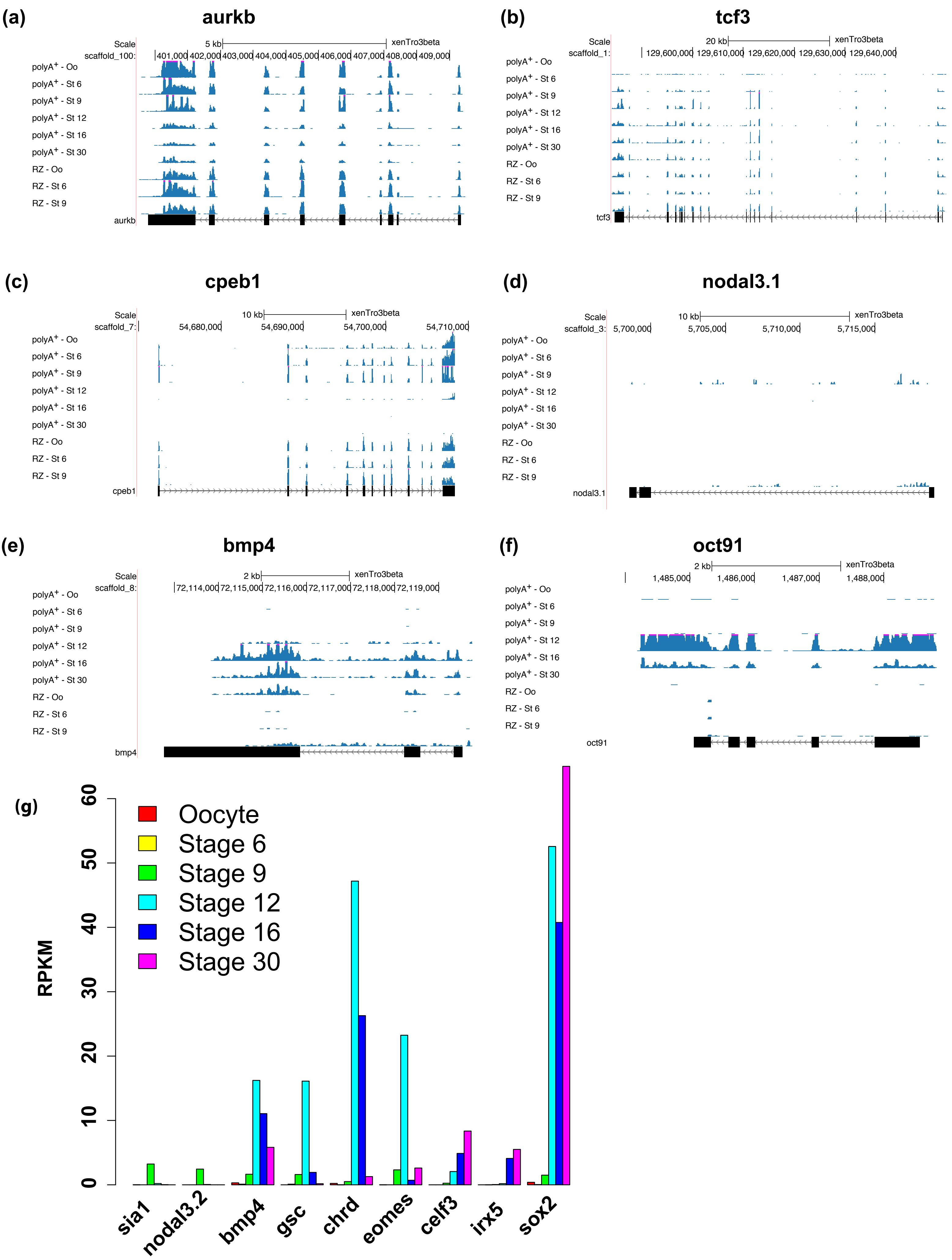

Figure S6. Flow-chart to describe *Xenopus tropicalis* Experimentally Validated Genes Annotation pipeline (v3.4)

Xenopus tropicalis Experimentally Validated Genes annotation pipeline

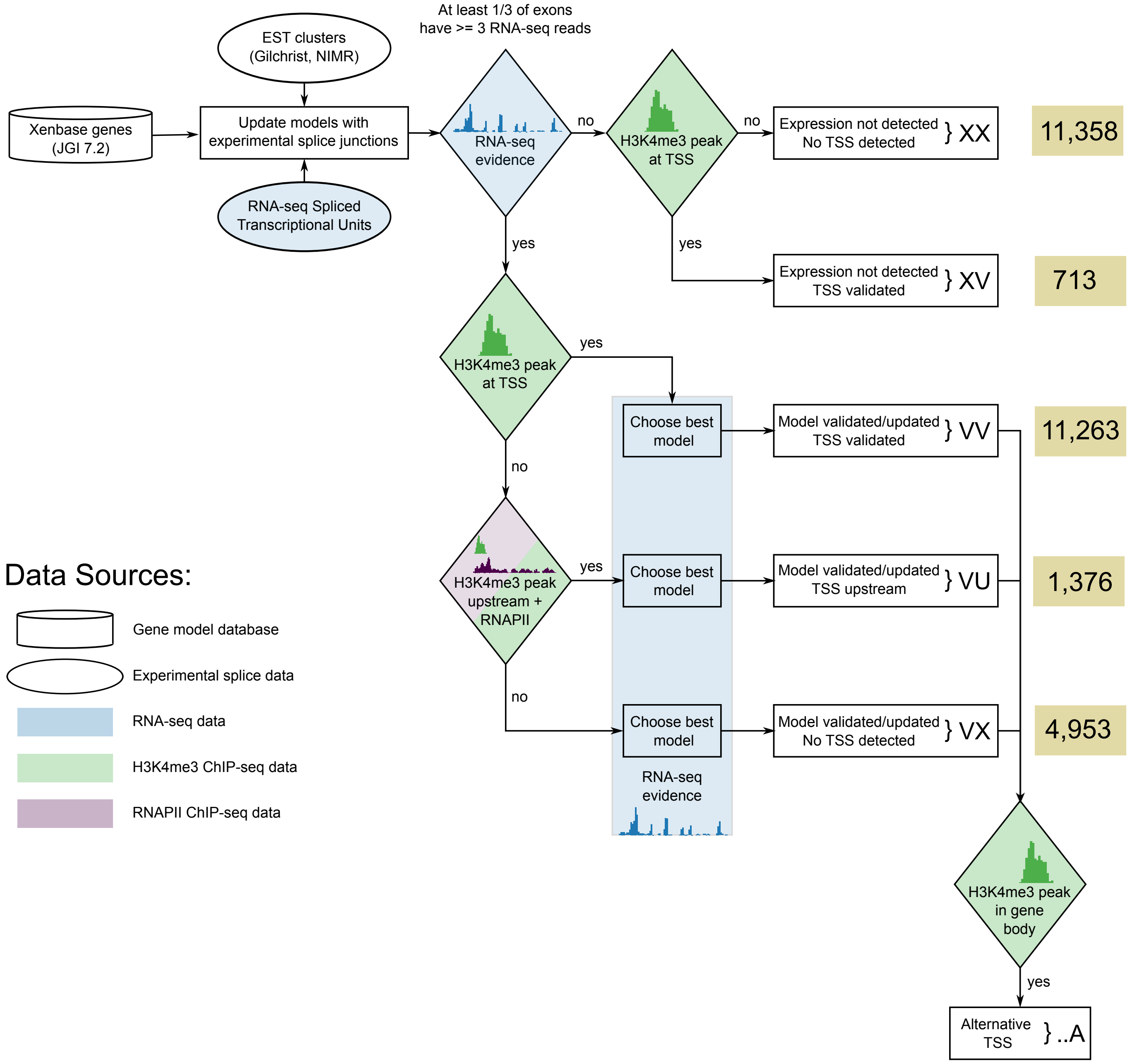

Gene Model Code:

V Validated, gene model with RNA-seq evidence

X No RNA-seq evidence, expression not detected

Xtev_2.198.3_VV(A) → (Optional) H3K4me3 peak in gene body, putative Alternative TSS

V Validated, H3K4me3 peak at TSS

U H3K4me3 peak Upstream, with RNAPII evidence

X No TSS evidence found

Figure S7.

(a) Statistics on updated Xtev(v3.4) gene models.

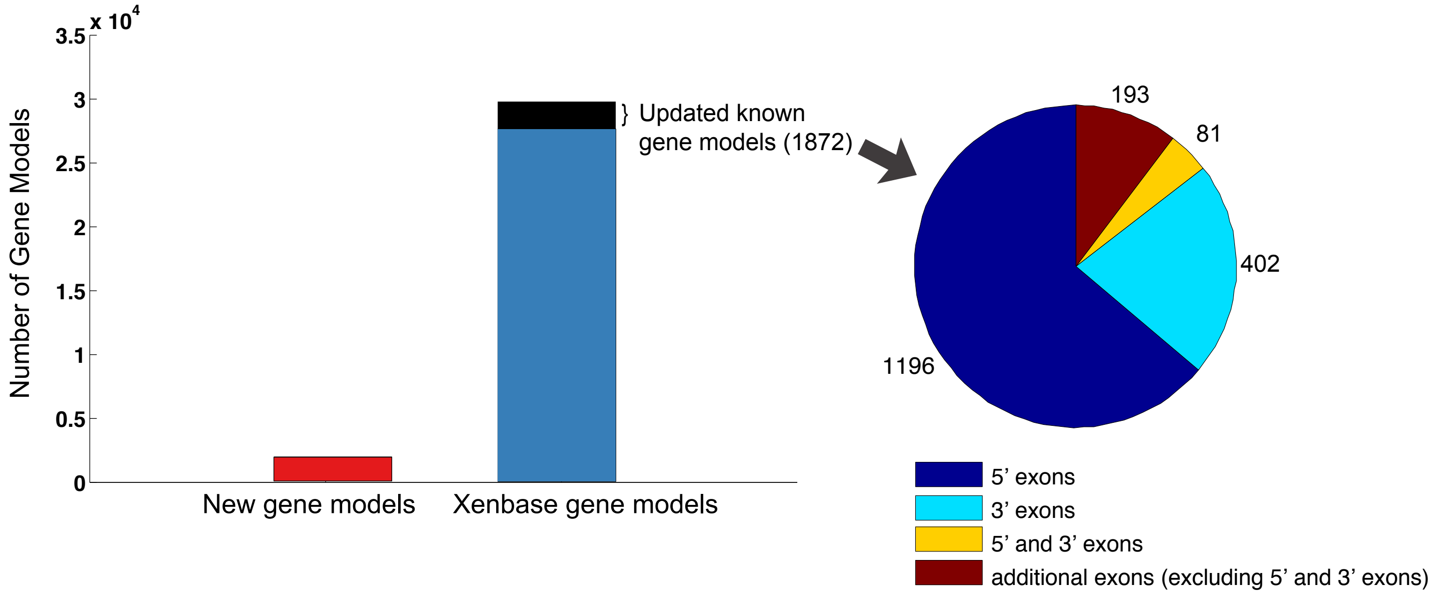

(b) MALAT1 : Highly conserved lncRNA from unspliced single exon gene models (CUFF.28619.1).
(for complete list of single exon gene models see Additional file 3)

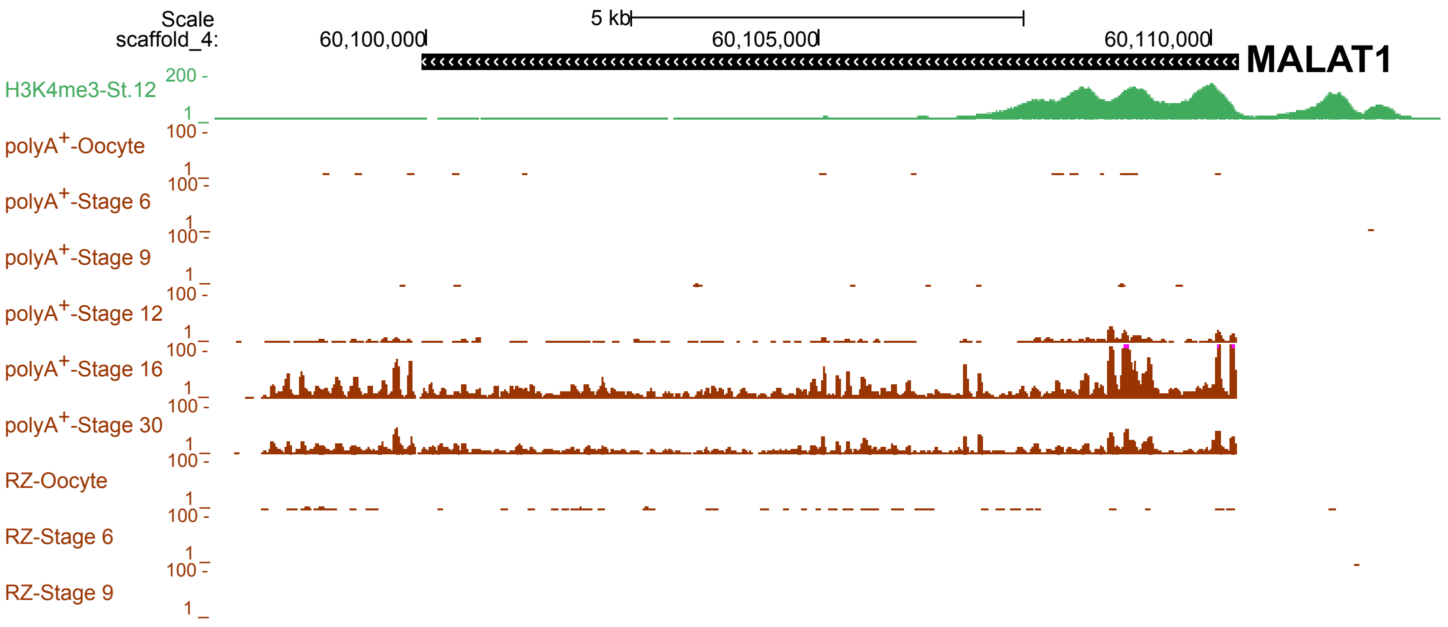

Figure S8

(a) Real-time qPCR validation of new gene models (NGM subset).

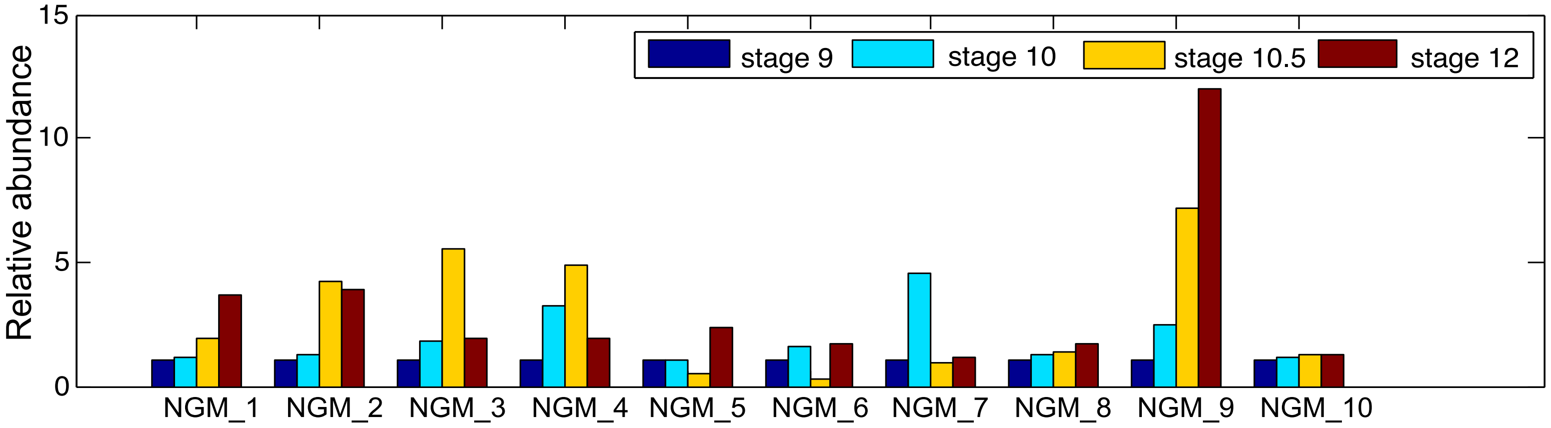

(b) Average polyA^{+} expression (RPKM, GM subset) profiles during embryogenesis.

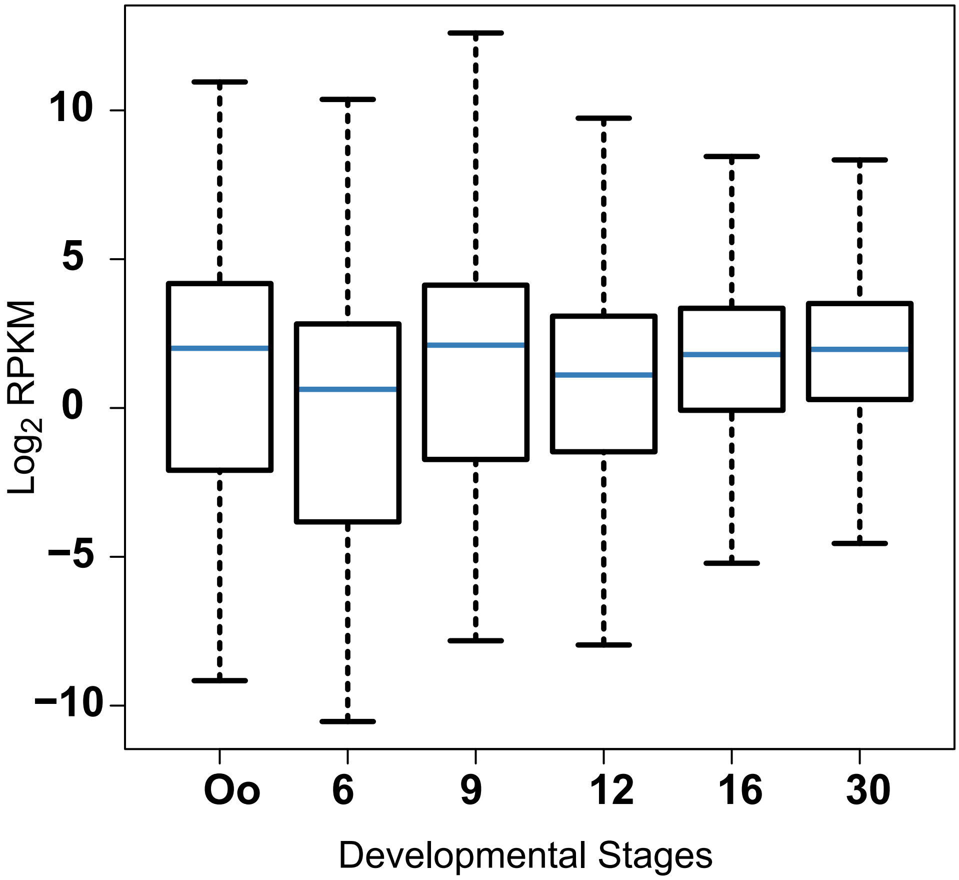

Figure S9

(a) Examples of lncRNAs from NGM_{VVO} subset.

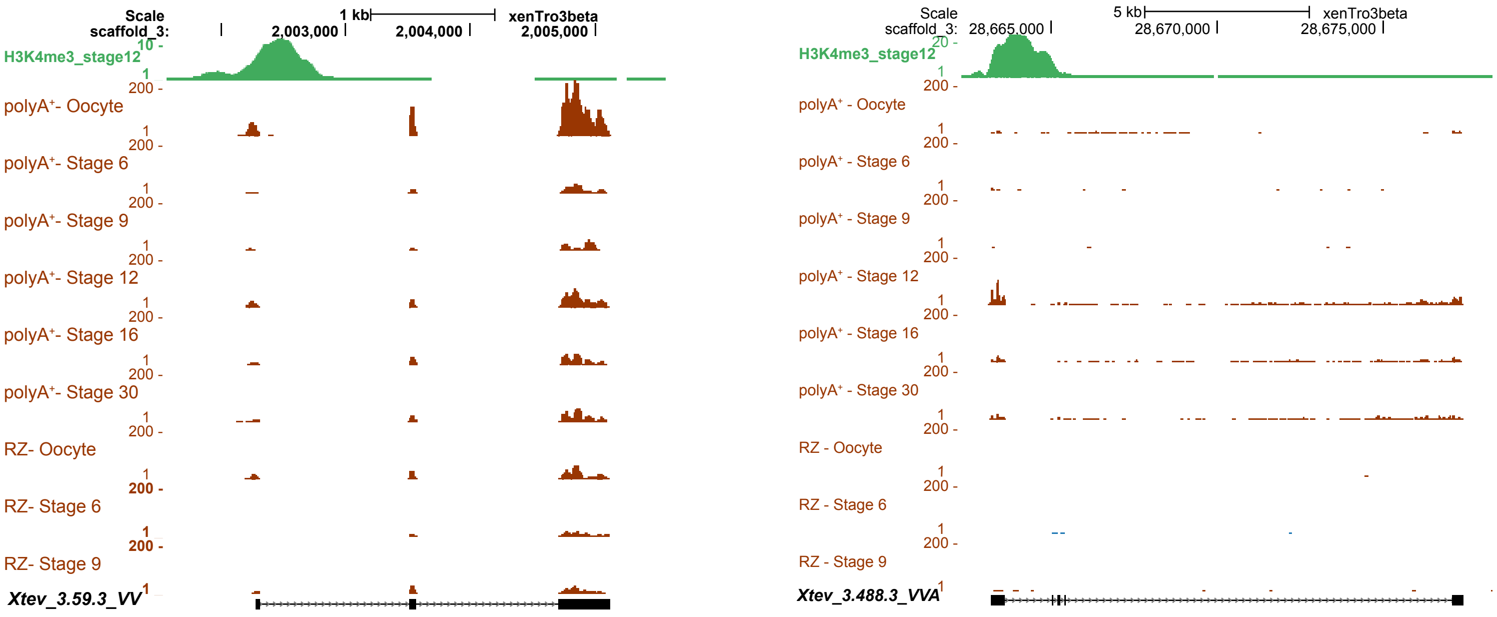

(b) Stage-specific $polyA^{+}$ expression (RPKM) of high-confidence lncRNAs (NGM_{VVO} subset).

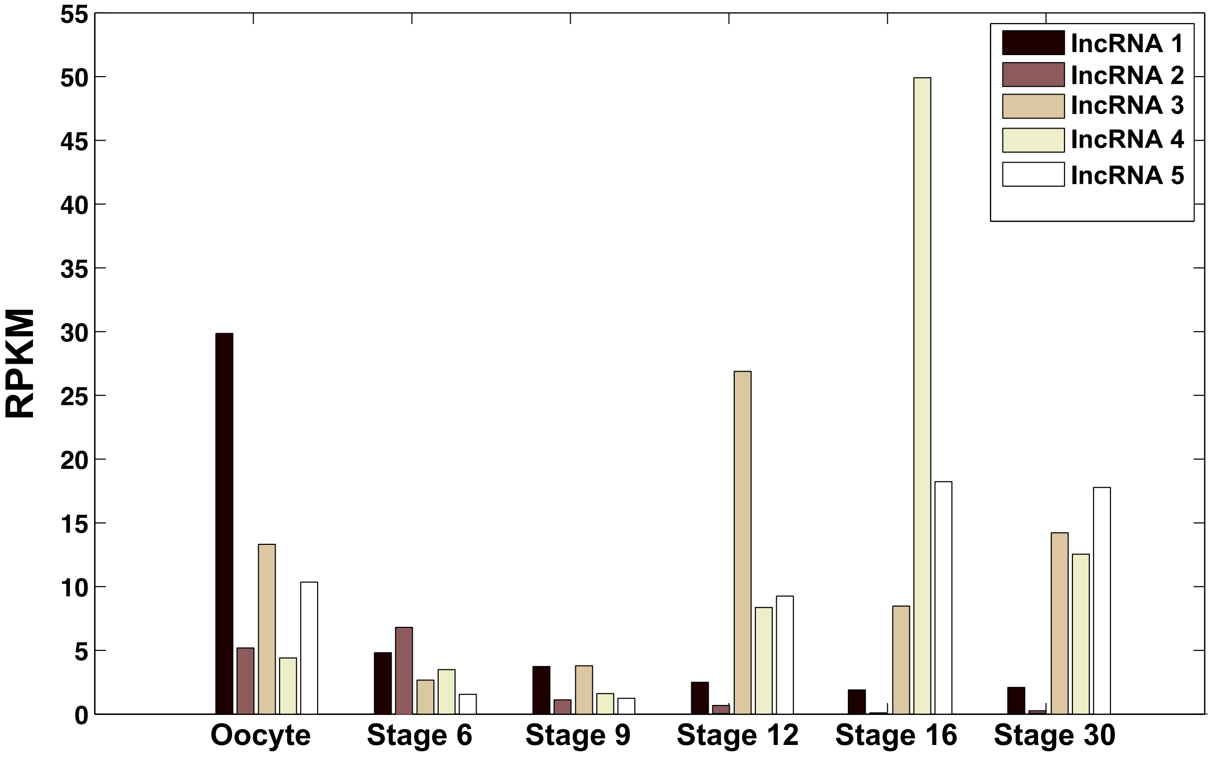

lncRNAs	NGM_{VVO} subset ID	Stage of peak expression
1	Xtev_9.872.3_VV	Oocyte
2	Xtev_2.453.3_VV	Stage 6
3	Xtev_3.488.3_VVA	Stage 12
4	Xtev_9.642.3_VV	Stage 16
5	Xtev_3.895.3_VV	Stage 30
